# Supplementary material for: Factors influencing implementation of an insulin patient decision aid at public health clinics in Malaysia: A qualitative study
Source: PLoS One. 2020 Dec 30;15(12):e0244645. doi: 10.1371/journal.pone.0244645 (PMC7773191; doi:10.1371/journal.pone.0244645)
Supplement: S1 Appendix — (PDF) [file pone.0244645.s001.pdf]

Appendix 1 Phase 1 interview guide for healthcare policy maker, healthcare providers and patients

DMIT3 INTERVIEW GUIDE:
HEALTHCARE POLICY MAKER

Preamble:
Ice-breaking
- Explain information given will be kept private and confidential
- Explain no right or wrong answer
- Need to get consent for the interview and recording
- Do not have to answer if the participant doesn't wish to do so
- Interested in getting a wide range of information and would appreciate if they can share their views as much as possible
- Explain that: *We would like to implement the insulin PDA in the public and university-based primary care clinics in Malaysia. This interview is about what are the barriers and facilitators to implementing the insulin PDA in your healthcare organization or in Malaysian public and university-based primary healthcare practices in general, so that it is effective and sustainable to be used.*
- **Show and tell on the 3 insulin PDA models**

General
1. What do you think about the insulin PDA?
   Probe: What is your feeling about introducing the use of the insulin PDA in your clinic/institution? (**example:** afraid that PDA will add to workload, hopeful, confuse, love). (emotions)
2. We would like to hear your honest opinion. Would you implement the PDA in your institution? Why? Why Not? (intention)
3. What are the barriers and facilitators you foresee in implementation of the insulin PDA in your institution?

*Interviewer to go to the respective section mentioned by respondent and probe on sections that were not brought up

The insulin PDA
4. What information do you/HCPs/patients need to know about the insulin PDA so you/HCPs/patient will use it? (**example**: objective, evidence strength, quality, the developer)
5. Which PDA model you think you would implement in your institution/clinic? Why?
   Probe: booklet, tablet, webpage

Outer context
6. How would you or your institution decide whether to implement/use the insulin PDA or not? (memory, attention and decision processes)
7. Can you think of an organizations or individuals that may influence your decision to implement the PDA? (**example:** Higher authority, patient, advocacy groups, NGOs, other countries). How do they influence the implementation of the insulin PDA? (social influences)
8. Will the implementation of the PDA result in any change in your organization or your healthcare system? Any benefit or harm in implementing the PDA? (**example:** positive or negative changes *-improve communication between HCP-Pt, reduce or prolong consultation time, provide information, facilitate doctor's consultation, administrative factors, additional resources, financial resources)* (intention, beliefs about consequences, optimism)

9. Are you confident/positive/optimistic that your institution is capable to successfully implement the insulin PDA despite any difficulty? Yes/No, why? (beliefs about capabilities, optimism)
10. *As a policy maker, what reinforcements you think can be done to ensure the successful implementation of the insulin PDA? (**example:** incentives, regulations, guidelines, reminders, protocols, audit, education and training, field visits, patient charters) (reinforcement)
11. If you will implement the insulin PDA in your organization, what are the goals you want to achieve? (**example:** *to develop professional expertise, to improve patient care)* (goals)
12. Do you think by implementing the insulin PDA, this will affect your organization/clinic's goals and targets? (**example:** Hba1c target, insulin uptake). How will it affect the implementation of the PDA? (social professional role and identity)

Inner context – The organization

13. Does your institution have the resources to implement the insulin PDA (booklet tablet, webpage)? What are the resources available and what are needed?
    Probe: time, technology support, infrastructure, spaces, staff, cost, working culture
14. *How can we address or enhance these environmental and resources barriers and facilitators?
15. How do you think your organization's working culture (general beliefs, values, assumptions that people embrace) will affect the implementation of the PDA? (environmental context and resources)
    Probe: How will they embrace the use of the insulin PDA in the clinic?

Individuals involved: healthcare policymaker, healthcare provider and patients

16. As a policy maker, how confident are you with the implementation of the insulin PDA? (**example:** the evidence, the institution which developed the insulin PDA) Why? (social professional role and identity)
17. Do you think HCPs/patients will want to use the insulin PDA? Why? (intentions)
18. *How can we increase HCPs'/patients' intention to use the PDA? (intentions)
19. *Who do you think will be using the insulin PDA with the patients or who will help to implement/coordinate the implementation of the insulin PDA? Why? (**example**: doctors, nurses, pharmacist) (social professional role and identity)
20. Do you think the HCPs/patients have the knowledge and skills to use the insulin PDA? (**example:** understanding of the insulin PDA, its purpose, and how to use, communication skills, computer skills, interpersonal skills, counselling skills, literacy skills, health conditions) (knowledge and skills)
21. *How can we improve HCPs/patients' knowledge and skills in order for them to use the insulin PDA effectively? (**example:** training, communication techniques, counselling techniques, continuing education, educational material, online information) (knowledge and skills)
22. How do you think HCPs/patients decide whether to use the PDA or not? (memory, attention and decision processes)
23. Do you think HCPs/patients would remember to use the PDA if it is implemented? Why? Why not? [**example:** too tired, too busy] (memory, attention and decision processes)
24. *What do you think can be done to make HCPs/patients to use the PDA?
    Probe: incentives, protocols, reminders, education and training, audit, laws monitoring system, field visits, patient charters, recommendations from regulatory body, peers, the medical community) (reinforcements)
25. What can HCPs/patient themselves do to ensure they use the insulin PDA? (behavioural regulation)
    Probe: self-change, undergo training, self-learning, breaking habit, action planning
26. Do you think HCPs/patients will be confident to use the insulin PDA? Why?

Probe: self-efficacy, self-commitment (beliefs about capabilities)

27. *How can we improve HCPs'/patient's <u>confidence</u> so they will use the insulin PDA? (beliefs about capabilities)
28. What <u>feelings</u> do you think HCPs will have if they were asked to use the insulin PDA (booklet, tablet, webpage)? (emotions; social professional role and identity)
29. Do you think the insulin PDA will affect HCP <u>role</u>? (**example:** losing control of the consultation, determining what is best for the patient, compromising clinical expertise and medical judgment). How will this affect the implementation of the PDA? (social professional role and identity)
30. What do you think patients will <u>feel</u> when you introduced the insulin PDA (booklet, tablet, webpage) to them? (example: afraid, fear, hopeful, sad, confuse).
    Probe: Do you think they will feel to be pushed to use insulin? How will this affect the implementation of the insulin PDA? (social professional role and identity)
31. What are the <u>resources</u> HCPs/patients need to use the PDA (booklet, tablet, webpage)? (environmental context and resources)
32. *How can these resources be provided to the HCPs/patients? (environmental context and resources)
33. <u>Who</u> do you think can influence HCPs/patient whether to use or not to use the insulin PDA? (**example**: colleagues, patients, higher authority) How? (social influences)

<u>Process of implementation*</u>

34. In your opinion, how the intervention can be integrated into your primary healthcare institution so that it is effective and sustainable?
35. How would you consider the intervention has achieved implementation success?
    Probe: How do you plan to assess the success level?

Lastly,

36. Could you share with us a programme that has been <u>implemented successfully</u> in your institution? Why?

37. Could you share with us a programme that has <u>failed to be successfully implemented</u> in your institution? Why?

Preamble:

Ice-breaking

- Explain information given will be kept private and confidential
- Explain no right or wrong answer
- Need to get consent for the interview and recording
- Do not have to answer if the participant doesn't wish to do so
- Interested in getting a wide range of information and would appreciate if they can share their views as much as possible
- Explain that: *We would like to implement the insulin PDA in the clinic here. We would like to hear your honest opinion on what are the barriers and facilitators to implementing the insulin PDA in your healthcare organization so that it is effective and sustainable to be used.*
- **Show and tell on the 3 insulin PDA models**

General

1. What do you think about the insulin PDA?
   Probe: How do you feel about the insulin PDA? (**example:** afraid that PDA will add to workload, hopeful, confuse, love). Do you like it? Why? Why not? (emotions)
2. Would you use this PDA? Why? Why Not? (intention, beliefs about consequences, optimism)
3. What are the barriers and facilitators you faced/foresee in implementation of the insulin PDA?

*Interviewer to go to the respective section mentioned by respondent and probe on sections that were not brought up

Insulin PDA

4. What information do you/patients need to know about the insulin PDA so you/patient will use it? (**example:** objective, evidence strength, quality, the developer)
5. Which PDA model you think is most feasible to be implemented in the clinic? Why?
   Probe: booklet, tablet, webpage

Individuals involved: healthcare provider and patients

Cognitive processes

6. Do you think HCPs'/patients will want to use the insulin PDA? Why? (intentions)
   Probe: What feelings will you/HCPs/patients have if you/they were asked to use the insulin PDA (booklet, tablet, webpage)?. (emotions; social professional role and identity) (**example:** afraid, fear, hopeful, sad, confuse, feel to be pushed to use insulin). How will this affect the implementation of the insulin PDA? (social professional role and identity)
7. Do you think the insulin PDA will affect your/HCP role? (**example:** losing control of the consultation, determining what is best for the patient, compromising clinical

expertise and medical judgment). How will this affect the implementation of the PDA? (social professional role and identity)

8. *How can we increase your/HCPs'/patients' intention to use the PDA? (intentions)
9. How do you/HCPs/patients decide whether to use the PDA or not? (memory, attention and decision processess)
10. Do you think you/HCPs/patients would remember to use the PDA if it is implemented? Why? Why not? [**example:** too tired, too busy] (memory, attention and decision processess)
11. Do you think you/HCPs/patients will be confident to use the insulin PDA? Why? *Probe:* self-efficacy, self-commitment (beliefs about capabilities)
12. *How can we improve your/HCPs'/patient's confidence so they will use the insulin PDA? (beliefs about capabilities)

Implementation processes

13. How doctors and nurses or other HCPs such as dieticians or pharmacists play a role in implementing the insulin PDA?
    Probe: Who do you think should be using the insulin PDA with the patients or who will help to implement/coordinate the implementation of the insulin PDA? Why? (**example**: doctors, nurses, pharmacist) (social professional role and identity)
14. Do you think the you/HCPS/patients have the knowledge and skills to use the insulin PDA (booklet, tablet, webpage)? (**example:** understanding of the insulin PDA, its purpose, and how to use, communication skills, computer skills, interpersonal skills, counselling skills, literacy skills, health conditions) (knowledge and skills)
15. *How can we improve your/HCPs/patients' knowledge and skills in order for them to use the insulin PDA effectively? (**example:** training, communication techniques, counselling techniques, continuing education, educational material, online information) (knowledge and skills)
16. Do you see any benefit or harm in implementing the PDA in the current healthcare system (booklet, tablet, webpage)? *(***example***: help in decision making, help to think about options, increase information seeking behaviours, improve communication between HCP-Pt, improve knowledge*) (beliefs about consequences)
17. Who do you think can influence you/HCPs/patient whether to use or not to use the insulin PDA? (**example**: colleagues, patients, higher authority, champion, opinion leader) How? (social influences)
18. *What do you think can be done to make you/HCPs/patients to use the PDA? *Probe:* incentives, protocols, reminders, education and training, audit, laws monitoring system, field visits, patient charters, recommendations from regulatory body, peers, the medical community) (reinforcements)
19. What can you/HCPs/patient your/themselves do to use the insulin PDA in their consultation? (behavioural regulation) *Probe:* self-change, undergo training, self-learning, breaking habit, action planning
20. If you will implement the insulin PDA, what are the goals you/patients want to achieve? (**example:** *to develop professional expertise, to improve patient care) (*goals)

Inner context – the clinic

21. Does your institution have the resources to implement the insulin PDA (booklet tablet, webpage)? What are the resources available and what are needed?
    Probe: infrastructure, time, technology support, spaces, staff, cost, working culture
22. *How can we address or enhance these environmental and resources barriers and facilitators?
23. How do you think your organization's working culture (general beliefs, values, assumptions that people embrace, receptivity of a new intervention) will affect the implementation of the PDA? (environmental context and resources)
    Probe: How will they embrace the use of the insulin PDA in the clinic?
24. If you use the PDA, will it result in any change in your clinic (**example:** positive or negative changes -*improve communication between HCP-Pt, reduce or prolong consultation time, provide information, facilitate doctor's consultation, administrative factors, additional resources, financial resources)* (beliefs about consequences)

Outer context – the healthcare system

25. Are you confident/positive/optimistic that the current healthcare system is capable to successfully implement the insulin PDA despite any difficulty? Yes/No, why? (beliefs about capabilities; optimism)
26. What can be done by the higher-level authority to ensure the success of the implementation of the PDA?
    Probe: national performance measures, policies, regulations, or guidelines (reinforcements)

Process of implementation*

27. In your opinion, how can we make the implementation of the insulin PDA sustainable?
28. How would you consider the intervention has achieved implementation success?
    Probe: How do you plan to assess the success?

Lastly,

29. Could you share with us a programme that has been implemented successfully in your institution? Why?

30. Could you share with us a programme that has failed to be successfully implemented in your institution? Why?

DMIT3 INTERVIEW GUIDE
PATIENTS

Preamble:
Ice-breaking
- Information given will be kept private and confidential
- Explain no right or wrong answer
- Need to get consent for the interview and recording
- Do not have to answer if the participant doesn't wish to do so
- Interested in getting a wide range of information and would appreciate if they can share their views as much as possible
- Explain that: *This interview is about what are the barriers and facilitators you think you or generally patients will face when receiving and using the insulin PDA. We also want to know from your opinion what are the best ways you want the PDA to be delivered to you/patients. Your input will help us to design an effective and innovative strategy for implementing the insulin PDA in primary care clinics in Malaysia.*

General

1. What do you think about the insulin PDA (booklet, tablet, webpage)?
   Probe: What are the feelings you have about the insulin PDA (booklet, tablet, webpage)? (**example**: feeling being pushed to use insulin, feeling bad having to start using insulin) (emotions) Do you like it? (booklet, tablet, webpage) Why? Why not? (emotions)
2. Would you/patients use the insulin PDA? Why? (intention)

We would like to implement the insulin PDA in the primary care clinics in Malaysia. You have been attending this clinic for your diabetes for some time and familiar with the system.

3. What are the barriers and facilitators you foresee if we want to implement the insulin PDA in the clinic? Do you think patients will use it?

*Interviewer to go to the respective section mentioned by respondent and probe on sections that were not brought up

Insulin PDA

4. What information do you/patients need to know about the insulin PDA so then only you/patients will use it? (**example:** objective, evidence strength, quality, the developer)
5. Which PDA model you think you/patients would most likely to use? Why?
   Probe: booklet, tablet, webpage
6. Would you/patients pay to use the insulin PDA? Who do you think should bear the cost for the insulin PDA? (Cost)

<u>Inner context – the clinic</u>

7. When do you want to receive the insulin PDA?
   Probe: Before, during or after consultation
8. Where do you think is the best place for you to use the PDA effectively? (**example**: clinic, home, etc). Why?
9. Do you think the clinic has the resources to implement the insulin PDA (booklet tablet, webpage)? What are the resources available and what are needed?
   Probe: infrastructure, time, technology support, spaces, staff, cost, working culture

<u>Individual involved</u>

<u>Knowledge, skills and beliefs about capabilities</u>

Healthcare provider

10. Do you think HCPs have the knowledge and skills to use the PDA with you/patients? Yes/no, why?
11. What are the knowledge and skills you think HCPs need to have to use the insulin PDA (booklet, tablet, webpage) with their patients? (**example:** understanding of the insulin PDA and its purpose (knowledge), computer skills, communication skills, interpersonal skills, counseling skills (skills)
12. How can we improve HCPs' skills so they can use the PDA (booklet, tablet, webpage) effectively? (**example***: skills training workshop).*
13. How doctors and nurses or other HCPs play a role in delivering the insulin PDA to patients?
    Probe: Who do you think is the most suitable to use the PDA with you/patients? Why? (**example**: doctor, nurse, pharmacist*)* (Social professional role and identity)

Patients

14. Which group of patients should be introduced this PDA? Why?
    Probe: Diabetes patients who require insulin, on oral medication but not yet require insulin?
15. Do you think you or most patients would be able to use the insulin PDA (booklet, tablet, webpage) on your/their own (without the help of a doctor/nurse/family member/caretaker)? Why/Why not? (knowledge and skills)
    Probe: If no, what kind of help do you need to use this PDA (booklet, tablet, webpage)? (**example:** understanding the purpose and content of the insulin PDA, how to use (knowledge) literacy skills, computer skills, health conditions (knowledge and skills)
16. What can the doctor/nurse/pharmacist do to help you/patients to use the PDA (booklet, tablet, webpage)?
17. You mentioned that you/patient would not be able to use the PDA (booklet, tablet, webpage) on your own. However, if help is provided, do you think you would be confident to use the PDA?' (Beliefs about capabilities - self efficacy)
18. How do you think we can increase your/patients' confidence in using the PDA (booklet, tablet, webpage)?

Social professional role and identity

19. How can you/patients help in the implementation of the insulin PDA? (Social professional role and identity)
20. Do you think the insulin PDA will affect you/patients in making decision for your diabetes treatment (or in the consultation with the doctor/nurse)? How? (**example**: confuses patient) (Social professional role and identity)
21. Do you think the insulin PDA will change your relationship with your doctor? How? and how will this affect you in using the insulin PDA? (Social professional role and identity; beliefs about consequences)

Social influences

22. Can you think of anyone or any organizations that may influence your/patients' decision to use the PDA? How do they influence you?
    *Probe***:** HCPs, family members, friends, other patients, support groups, NGOs.

Intentions, memory, attention and decision processes, reinforcement, behavioural regulations)

23. Is there anything that would make you want to use the PDA? (memory, attention and decision process)
    Probe: *incentives, reminders, recommendations from HCP, peer education, advertise the PDA on TV)* (Reinforcements)
24. Do you think you/patients would remember to use the PDA? Why? Why not? [**example:** too tired, too busy] (memory, attention and decision process)
25. What can you/patients yourself/themselves do to use the insulin PDA (**Behavioural regulation**: **(example:** self-change, undergo training, self-learning, breaking habit, action planning) (Behavioural regulation)

Optimism, intentions, beliefs about consequences and goals

26. Are you positive/optimistic that the PDA can help you in making decision on insulin initiation? Why? (**example:** the content and evidence, the developer) (optimism)
27. Do you see any benefit or harm in using the PDA? *(***example***: help in decision making, help to think about options, increase information seeking behaviours, improve communication between HCP-Pt, improve knowledge*) (beliefs about consequences)
28. If you are going to use the PDA, what do you would want to achieve? (goals)

Outer context – the healthcare system

29. Do you think our country healthcare system is capable to successfully implement the insulin PDA in all the health clinics despite any difficulty? Yes/No, why? (beliefs about capabilities; optimism)
30. What do you think our country's healthcare system can do to ensure the success of the implementation of the PDA in all the health clinics?
    Probe: national performance measures, policies, regulations, or guidelines (enforcements)

Process of implementation*

31. In your opinion, how can we make the implementation of the insulin PDA sustainable in the clinic?
32. How would you consider the intervention has achieved implementation success?
    Probe: How do you plan to assess the success?

Lastly,

33. Could you share with us a programme that you felt has been successfully implemented? Why?
34. Could you share with us a programme that you felt has failed to be successfully implemented? Why?
