# Supplementary material for: Factors influencing implementation of an insulin patient decision aid at public health clinics in Malaysia: A qualitative study
Source: PLoS One. 2020 Dec 30;15(12):e0244645. doi: 10.1371/journal.pone.0244645 (PMC7773191; doi:10.1371/journal.pone.0244645)
Supplement: S2 Appendix — (DOCX) [file pone.0244645.s002.docx]

S2 Appendix: Detailed information of individual participant in each clinic

| **Inter-view type** | **Interviewer** | **Participant ID** | **Age** | **Ethnicity** | **Highest education level** | **HCP duration of practice at clinic (years)** | **Patient duration of seeking diabetes treatment in the clinic (years)** | **Patient’s occupation** | **Patient insulin use** | **Patient duration of insulin use (years)** |
| --- | --- | --- | --- | --- | --- | --- | --- | --- | --- | --- |
| **Clinic A** | | | | | | | | | | |
| IDI 1 | YKL | IDI 1_Clinic manager | 49 | Chinese | Postgraduate | 4 | - | - | - | - |
| IDI 2 | WTT | IDI 2_Patient 1 | 76 | Chinese | Secondary | - | 6 | Retiree | No | -* |
| IDI 3 | WTT | IDI 3_Patient 2 | 70 | Indian | Secondary | - | 6 | Retiree | Yes | 3 |
| IDI 4 | YKL | IDI 4_Patient 3 | 61 | Chinese | Secondary | - | 4 | Retiree | Yes | 4 |
| FGD 1 | WTT | FGD 1_MO 1 | 31 | Indian | Undergraduate | 1 | - | - | - | - |
|  |  | FGD 1_MO 2 | 35 | Indian | Postgraduate | 2 | - | - | - | - |
| FGD 2 | YKL | FGD 2_Diabetes educator | 36 | Malay | Postgraduate | 4 | - | - | - | - |
|  |  | FGD 2_Pharmacist | 29 | Malay | Undergraduate | 5 | - | - | - | - |
| **Clinic B** | | | | | | | | | | |
| IDI 5 | WTT | IDI 5_Clinic manager | 49 | Malay | Postgraduate | 5 | - | - | - | - |
| IDI 6 | YKL | IDI 6_MO 1 | 43 | Chinese | Undergraduate | 6 | - | - | - | - |
| IDI 7 | WTT | IDI 7_Pharmacist | 28 | Malay | Postgraduate | 3 | - | - | - | - |
| IDI 8 | YKL | IDI 8_Diabetes educator | 30 | Malay | Postgraduate | 4 | - | - | - | - |
| IDI 9 | WTT | IDI 9_MO 2 | 35 | Indian | Undergraduate | 7 months | - | - | - | - |
| FGD 3 | WTT | FGD 3_Patient 1 | 42 | Malay | Undergraduate | - | 11 | Human resource officer | Yes | 10 |
|  |  | FGD 3_Patient 2 | 36 | Malay | Secondary | - | 5 | City hall worker | Yes | 12 |
| **Clinic C** | | | | | | | | | | |
| IDI 10 | WTT | IDI 10_Patient 1 | 65 | Malay | Secondary | - | 3 | Housewife | Yes | 3 |
| FGD 4 | WTT | FGD 4_Clinic manager | 48 | Malay | Postgraduate | 11 months | - | - | - | - |
|  |  | FGD 4_MO 1 | 32 | Malay | Undergraduate | 3 | - | - | - | - |
|  |  | FGD 4_Pharmacist | 29 | Chinese | Undergraduate | 1 | - | - | - | - |
| FGD 5 | WTT | FGD 5_Diabetes educator | 36 | Malay | Postgraduate | 10 | - | - | - | - |
|  |  | FGD 5_MO 2 | 31 | Malay | Undergraduate | 2 months | - | - | - | - |
| FGD 6 | WTT | FGD 6_Patient 2 | 62 | Malay | Secondary | - | 5 | Retiree | Yes | 6 months |
|  |  | FGD 6_Patient 3 | 62 | Malay | Secondary | - | 2 months | Driver | No | -* |
|  |  | FGD 6_PatIent 4 | 58 | Malay | Undergraduate | - | 6 | Teacher | No | -* |
| **Clinic D** | | | | | | | | | | |
| IDI 11 | WTT | IDI 11_Clinic manager | 59 | Malay | Postgraduate | 1 | - | - | - | - |
| IDI 12 | YKL | IDI 12_MO 1 | 42 | Indian | Postgraduate | 10 | - | - | - | - |
| IDI 13 | WTT | IDI 13_Family medicine specialist | 41 | Malay | Postgraduate | 3 | - | - | - | - |
| IDI 14 | WTT | IDI 14_Patient 1 | 58 | Indian | Secondary | - | 10 | Retiree | Yes | 3 |
| IDI 15 | WTT | IDI 15_Patient 2 | 59 | Malay | Primary | - | 8 | Tea lady | Yes | 2 |
| FGD 7 | WTT | FGD 7_Staff nurse 1 | 34 | Indian | Undergraduate | 11 | - | - | - | - |
|  |  | FGD 7_Pharmacist 1 | 29 | Chinese | Undergraduate | 4 | - | - | - | - |
|  |  | FGD 7_Staff nurse 2 | 29 | Malay | Postgraduate | 4 | - | - | - | - |
|  |  | FGD 7_Staff nurse 3 | 40 | Malay | Undergraduate | 1 year 2 months | - | - | - | - |
|  |  | FGD 7_Staff nurse 4 | 44 | Malay | Postgraduate | 13 | - | - | - | - |
| FGD 8 | YKL | FGD 8_Patient 3 | 53 | Malay | Primary | - | 20 | Cleaner | Yes | 11 |
|  |  | FGD 8_Patient 4 | 47 | Malay | Primary | - | 19 | Seller | Yes | 11 |
| **Clinic E** | | | | | | | | | | |
| IDI 16 | WTT | IDI 16_Pharmacist 1 | 31 | Indian | Undergraduate | 5 | - | - | - | - |
| IDI 17 | YKL | IDI 17_Clinic manager | 54 | Indian | Postgraduate | 9 | - | - | - | - |
| IDI 18 | YKL | IDI 18_Patient 1 | 54 | Malay | Secondary | - | 7 | Not working | Yes | 4 |
| IDI 19 | WTT | IDI 19_Patient 2 | 37 | Malay | Undergraduate | - | 4 | Programmer | Yes | 2 |
| FGD 9 | WTT | FGD 9_MO 1 | 32 | Indian | Undergraduate | 1 year 9 months | - | - | - | - |
|  |  | FGD 9_Pharmacist | 29 | Malay | Undergraduate | 4 | - | - | - | - |
|  |  | FGD 9_Staff nurse 1 | 30 | Indian | Undergraduate | 1 | - | - | - | - |
