# Supplementary material for: Factors influencing implementation of an insulin patient decision aid at public health clinics in Malaysia: A qualitative study
Source: PLoS One. 2020 Dec 30;15(12):e0244645. doi: 10.1371/journal.pone.0244645 (PMC7773191; doi:10.1371/journal.pone.0244645)
Supplement: S1 Checklist — (DOCX) [file pone.0244645.s003.docx]

**Consolidated criteria for reporting qualitative studies (COREQ): 32-item checklist**

Developed from:

Tong A, Sainsbury P, Craig J. Consolidated criteria for reporting qualitative research (COREQ): a 32-item checklist for interviews and focus groups. *International Journal for Quality in Health Care*. 2007. Volume 19, Number 6: pp. 349 – 357

**YOU MUST PROVIDE A RESPONSE FOR ALL ITEMS. ENTER N/A IF NOT APPLICABLE**

| **No. Item** | **Guide questions/description** | **Reported on Page #** |
| --- | --- | --- |
| **Domain 1: Research team and reﬂexivity** |  |  |
| *Personal Characteristics* |  |  |
| 1. Inter viewer/facilitator | Which author/s conducted the interview or focus group? | Page 11, line 161-164  WTT conducted most of the interviews (13/19 IDIs; 7/9 FGDs). To ensure the study rigour, YKL, CJN and PYL were refrained from conducting interviews. However, YKL conducted a few IDIs and FGDs when some of the interviews had to be carried out concurrently. |
| 2. Credentials | What were the researcher’s credentials? E.g. PhD, MD | Page 10, line 138-139  The researchers of this study are WTT (MMedSc), YKL (PhD), NCJ (MBBS, PhD) and PYL (MBBS, MMed). |
| 3. Occupation | What was their occupation at the time of the study? | Page 10, line 139-140:  WTT is a PhD student while YKL, CJN and PYL are lecturers. CJN and PYL are also clinicians who specialize in family medicine. |
| 4. Gender | Was the researcher male or female? | N/A:  The researchers felt that their gender may not have significant impact on the study findings given that the topic of this study is one that is not gender-sensitive. |
| 5. Experience and training | What experience or training did the researcher have? | Page 10, line 140-141  All researchers are experienced in conducting qualitative research. |
| *Relationship with participants* |  |  |
| 6. Relationship established | Was a relationship established prior to study commencement? | Page 10, line 142-146  Prior to this research, YKL, CJN and PYL have collaborated with clinic manager C in the development of the insulin PDA thus clinic manager C was aware of the availability of the insulin PDA. Other clinic managers knew YKL, CJN and PYL given that they were all in the field of family medicine but only at the level of acquaintance. |
| 7. Participant knowledge of the interviewer | What did the participants know about the researcher? e.g. personal goals, reasons for doing the research | Page 10, line 145-146  Other clinic managers knew YKL, CJN and PYL given that they were all in the field of family medicine but only at the level of acquaintance.  Page 11, line 169-172  Prior to the interviews, the researchers gave the study information sheet and explained to the participants the purpose of the study, information on the insulin PDA, the concept of SDM and the various PDA modalities (booklet, tablet, website) available before informed consent was obtained from the participants. |
| 8. Interviewer characteristics | What characteristics were reported about the inter viewer/facilitator? e.g. Bias, assumptions, reasons and interests in the research topic | Page 10-11, line 142-167  **Prior to this research, YKL, CJN and PYL have collaborated with clinic manager C in the development of the insulin PDA thus clinic manager C was aware of the availability of the insulin PDA. Other clinic managers knew YKL, CJN and PYL given that they were all in the field of family medicine but only at the level of acquaintance.** All clinic managers agreed to participate and site meetings were set to know more about the clinic context as well as provide more information to the clinic managers. During the site meetings, WTT and YKL were brought around the sites and explained on the clinics’ running processes. Through this, the researchers were able to observe and understand each of the clinic contexts better. Then, the clinic managers were subsequently asked to refer researchers to staff who fulfilled the participant inclusion criteria for this study. In majority of the clinics, all the HCPs in the diabetes team were referred for participation, as they were the most relevant individuals except for Clinic C, which was without a diabetes team. The clinic manager of Clinic C selected the HCPs to participate. Appointments were then made with individuals who agree to participate to conduct the interviews. Patient participants were recruited by seeking help from the medical officers who were practising in the clinic on the day the interviews were conducted with the clinic managers and the HCPs. Participant response rate was 100% for both HCPs and patients. All the interviews were conducted in the clinics for the convenience of the participants. Only the interviewer and the interviewees were present during the interviews. No repeat interviews were conducted. WTT conducted most of the interviews (13/19 IDIs; 7/9 FGDs). To ensure the study rigour, YKL, CJN and PYL were refrained from conducting interviews. However, YKL conducted a few IDIs and FGDs when some of the interviews had to be carried out concurrently. **Being the developers of the insulin PDA, YKL, CJN and PYL were aware of their own biases and tried their best to distance their personal judgments when carrying out the data collection and analysis.** |
| **Domain 2: study design** |  |  |
| *Theoretical framework* |  |  |
| 9. Methodological orientation and Theory | What methodological orientation was stated to underpin the study? e.g. grounded theory, discourse analysis, ethnography, phenomenology, content analysis | Page 6, line 81-93  **This study adopted a comparative case study design with a qualitative focus** [32, 33]. This study design adopts similar approach as qualitative case study design whereby a phenomenon is studied in-depth by taking consideration of its context [32]. However, comparative case study design also allows understanding of similarities and differences across cases and is useful for understanding how context can influences the success of an intervention [32]. This study design was chosen because it allowed identification of similarities and differences of potential barriers and facilitators to implementing the insulin PDA across different implementation sites. It also enables identification of unique barriers and facilitators for individual sites. This can help inform development of common implementation strategies for several sites as well as tailored implementation strategy for individual sites for optimal implementation. The adoption of qualitative design allows in-depth understanding of the barriers and facilitators to implementation of the insulin PDA within and across the implementation contexts.  Page 12, line 192  **Thematic analysis** was conducted. |
| *Participant selection* |  |  |
| 10. Sampling | How were participants selected? e.g. purposive, convenience, consecutive, snowball | Page 9, line 111  The participants of this study were **purposively sampled**… |
| 11. Method of approach | How were participants approached? e.g. face-to-face, telephone, mail, email | Page 10, line 142-158  WTT sent **e-mail invitations** to clinic managers to participate in the study. Prior to this research, YKL, CJN and PYL have collaborated with clinic manager C in the development of the insulin PDA thus clinic manager C was aware of the availability of the insulin PDA. Other clinic managers knew YKL, CJN and PYL given that they were all in the field of family medicine but only at the level of acquaintance. All clinic managers agreed to participate and site meetings were set to know more about the clinic context as well as provide more information to the clinic managers. During the site meetings, WTT and YKL were brought around the sites and explained on the clinics’ running processes. Through this, the researchers were able to observe and understand each of the clinic contexts better. Then, the clinic managers were subsequently asked to refer researchers to staff who fulfilled the participant inclusion criteria for this study. In majority of the clinics, all the HCPs in the diabetes team were referred for participation, as they were the most relevant individuals except for Clinic C, which was without a diabetes team. The clinic manager of Clinic C selected the HCPs to participate. **Appointments were then made using telephone with individuals who agree to participate to conduct the interviews.** **Patient participants were recruited face-to-face** by seeking help from the medical officers who were practising in the clinic on the day the interviews were conducted with the clinic managers and the HCPs. |
| 12. Sample size | How many participants were in the study? | Page 14, line 231-233  A total of 19 IDIs and 9 FGD were conducted with a range of **43 stakeholders (policymaker: 5; doctor: 9; pharmacist: 6; diabetes educator: 3; pharmacist: 6; patients: 15)** from the five clinics. |
| 13. Non-participation | How many people refused to participate or dropped out? Reasons? | Page 11, line 159  Participant response rate was 100% for both HCPs and patients. |
| *Setting* |  |  |
| 14. Setting of data collection | Where was the data collected? e.g. home, clinic, workplace | Page 11, line 159-160  All the interviews were conducted in the clinics for the convenience of the participants |
| 15. Presence of non-participants | Was anyone else present besides the participants and researchers? | Page 11, line 160-162  Only the interviewer and the interviewees were present during the IDI while for FGDs, a note taker was also present to help capture verbatim notes. |
| 16. Description of sample | What are the important characteristics of the sample? e.g. demographic data, date | Page 15  Table 2: Participants’ socio-demographic information by clinic  Appendix 2: Detailed information of individual participant in each clinic |
| *Data collection* |  |  |
| 17. Interview guide | Were questions, prompts, guides provided by the authors? Was it pilot tested? | Page 9-10, line 133-135  Questions and prompts were created according to the domains in the TDF and adapted according to healthcare policymaker, HCPs and patient participants (Appendix 1).  Was it pilot tested?  N/A  Reason: The interview guide was not piloted tested, however, the researchers discussed on the relevance of the questions and prompts and ensured all the domains in the Theoretical Domains Framework were covered. In addition, the researchers adopted a flexible approach when using the interview guide allowing changes to be made in terms of the questions asked depending on interviewees’ responses. |
| 18. Repeat interviews | Were repeat inter views carried out? If yes, how many? | Page 11, line 161  No repeat interviews were conducted. |
| 19. Audio/visual recording | Did the research use audio or visual recording to collect the data? | Page 11, line 182-183  All the interviews were audio-recorded and they lasted on average 50-90 minutes. |
| 20. Field notes | Were ﬁeld notes made during and/or after the inter view or focus group? | Page 11-12, line 182-188  Field notes were taken to capture data that cannot be audio-recorded such as observations on the clinic surrounding and participants’ non-verbal gestures. Interviews ceased when there was no new information that emerged from the participants in each of the clinic (data saturation). In addition, interview reflections were also noted at the end of each interview sessions to capture researchers’ views about the interview sessions as well as the points that has been raised by the participants. |
| 21. Duration | What was the duration of the inter views or focus group? | Page 11, line 182-183  All the interviews were audio-recorded and they lasted on average 50-90 minutes. |
| 22. Data saturation | Was data saturation discussed? | Page 12, line 184-186  Interviews ceased when there was no new information that emerged from the participants in each of the clinic (data saturation). |
| 23. Transcripts returned | Were transcripts returned to participants for comment and/or correction? | No.  Reason: All the audio recordings were transcribed verbatim and checked by the researcher to ensure that all the narratives that occurred during the interviews were included. |
| **Domain 3: analysis and ﬁndings** |  |  |
| *Data analysis* |  |  |
| 24. Number of data coders | How many data coders coded the data? | Page 13-14, line 202-216  The data analysis was performed case by case for each clinic at a time in order to gain in-depth understanding on the contextual factors that influence implementation of the insulin PDA at the specific setting. **Initially, the researchers WTT and YKL read the transcripts in detailed from Clinic A and coding was performed independently**. Categories and themes emerged were discussed and finalized when discrepancies were resolved. The finalized themes and categories for Clinic A were later used as a coding framework for data analysis for the rest of the clinics. Any new codes and categories that emerged were added to the coding framework while those that were not relevant were removed. **WTT coded all the transcripts for all the clinics while YK coded for Clinic B and C, PYL coded for Clinic C and clinic D and CJN coded for Clinic E, independently.** Align with the comparative case approach, which used various forms of data [32], field notes from the sites observations and interview reflections were also referred to help in understanding and interpretation of the data. Furthermore, data analysis were also performed sequentially starting from Clinic A to Clinic E that allowed WTT to fully immerse into the data for each clinic as well as making comparisons to other clinics as categories and themes emerged [32]. |
| 25. Description of the coding tree | Did authors provide a description of the coding tree? | Page 12-13  Table 1 shows the development of the coding frame. |
| 26. Derivation of themes | Were themes identiﬁed in advance or derived from the data? | Page 12, line 193-209  Initially, transcripts were read line by line and codes (short phrases label) were assigned to specific data sections that represented their significance (open coding). Then, codes that were developed were reviewed and group together to form categories (axial coding). Related categories were then reviewed and overarching themes were applied that reflected the meaning of the data. Once the coding framework was developed, it was then used to code data from other transcripts (selective coding) [40].  The data analysis was performed case by case for each clinic at a time in order to gain in-depth understanding on the contextual factors that influence implementation of the insulin PDA at the specific setting. Initially, the researchers WTT and YKL read the transcripts in detailed from Clinic A and coding was performed independently. Categories and themes emerged were discussed and finalized when discrepancies were resolved. The finalized themes and categories for Clinic A were later used as a coding framework for data analysis for the rest of the clinics. Any new codes and categories that emerged were added to the coding framework while those that were not relevant were removed. |
| 27. Software | What software, if applicable, was used to manage the data? | Page 12, line 191-192  The interviews were transcribed verbatim and checked by the researcher (WTT) before imported into **NVivo qualitative software for analysis.** |
| 28. Participant checking | Did participants provide feedback on the ﬁndings? | The findings from the data analysis were not provided to the participants for feedback. However, the researcher ensured that the data were accurately interpreted by crosschecking the analysis of the findings to interview reflections and memos that were jotted down at the end of each interview.  Nevertheless, we have now included the absence of participant checking in the study limitations |
| *Reporting* |  |  |
| 29. Quotations presented | Were participant quotations presented to illustrate the themes/ﬁndings? Was each quotation identiﬁed? e.g. participant number | Page 17-23  Results section |
| 30. Data and ﬁndings consistent | Was there consistency between the data presented and the ﬁndings? | Page 17-23  Results section |
| 31. Clarity of major themes | Were major themes clearly presented in the ﬁndings? | Page 17, line 236-239  Data analysis uncovered five themes that were perceived as potential factors that could influence the implementation of the insulin PDA in the five clinics and they were: time, patient load and lack of manpower; cost to print the PDA booklets; tailoring PDA use to patient profile; patient decisional role; and leadership and staff motivation. |
| 32. Clarity of minor themes | Is there a description of diverse cases or discussion of minor themes? | N/A. No minor themes or diverse cases emerged from the data analysis. |

**Once you have completed this checklist, please save a copy and upload it as part of your submission. When requested to do so as part of the upload process, please select the file type: *Checklist*. You will NOT be able to proceed with submission unless the checklist has been uploaded. Please DO NOT** **include this checklist as part of the main manuscript document. It must be uploaded as a separate file.**
