# Supplementary material for: Factors influencing implementation of an insulin patient decision aid at public health clinics in Malaysia: A qualitative study
Source: PLoS One. 2020 Dec 30;15(12):e0244645. doi: 10.1371/journal.pone.0244645 (PMC7773191; doi:10.1371/journal.pone.0244645)
Supplement: Attachment — Submitted filename: Response to reviewers.docx [file pone.0244645.s004.docx]

|  | **Reviewer #1** | **Response to reviewer** |
| --- | --- | --- |
| 1. | The real challenge, as implementation of Patient Decision Aids is not a goal in itself, is the implementation of a true dialogue and sharing of decision making attributes, values and preferences between doctor and patient. The authors could reflect on this overarching aim in the discussion. | **Response to reviewer**  We thank the reviewer for the suggestion and have now highlighted the point on the importance of PDA in facilitating true dialogue and sharing of decision making attributes, values and preferences between doctor and patient in the Discussion section.  **Page 27-28, line 484-521**  ***This study highlighted numerous potential challenges of enabling PDA to be implemented in the clinic, however, it should be noted that PDA use does not guarantee that SDM conversation occurs between doctor and patient.*** *The HCPs of this study highlighted that some patients tend to rely on others to make health decisions for them. Such beliefs may influence HCPs not to use PDA with patients due to the perception that a patient would not be interested or able to participate in SDM [54]. A study on unvoiced needs in five Malaysian public health clinics shows that 26.7% of patients did not express their concerns to healthcare provider during clinical encounter [55]. Among the reasons were patients felt embarrassed and nervous to ask HCPs, expected HCPs to enquire, and forgot to ask [56]. The findings of our study together with Low et al.’s (2009) study indicate that efforts to increase patients’ awareness on being more involved in their health care and self-efficacy in making health decision, as well as participation in SDM is warranted. The first step that can be taken is to encourage patients to ask HCPs questions about their health. Low et al., (2009) has developed an intervention to reduced patients’ unvoiced needs by getting patients to note down issues to be discussed with doctors prior to their clinical encounter, making patients’ and HCPs to be aware of the issue of unvoiced needs through usage of video, poster, pamphlets, note books and stick-on notes. The interventions have been shown to be effective [56] and may be adapted to faciliate the implementation of the insulin PDA.*  ***Apart from empowering patients, HCPs also need to be taught on the concept of SDM. Based on the qualitative narratives, it can be found that some HCPs might not had been able to fully grasp the concept of SDM (Theme 4, quote 1: “Shared decision making is mainly letting the patient to make the decision themselves…”) in the short presentation, which was given prior to the interview. HCPs need to understand their role in SDM in terms of providing quality information to patients and supporting patients in their deliberation of the treatment option [1] rather than placing the onus of health decision making to patients. SDM is generally a foreign concept in Malaysia in that SDM activities only began in 2010 [57]. While the Ministry of Health Malaysia and the Malaysian Medical Council has encourage doctors to perform SDM-related activities such as providing patients with accurate and timely information, discuss treatment limitation and risks, and collaborate with patient in selection of treatment since 1990s [57-59], patient involvement in healthcare decision making in Malaysia was still found to be lacking as there is no formal training and education, laws, regulations and health policies that supports SDM [65]. SDM is not taught explicitly in the medical undergraduate and postgraduate curriculum [57]. To implement SDM and PDA effectively, HCP training is warranted. Studies have reported the lack of training to be contributing factors to lack of engagement of patients in SDM and proper use of PDAs [66, 67]. The benefits of training are well evidenced [60, 61] and it should be a pre-requisite for SDM and PDA implementation [68].*** |
| 2. | Methods  It is not clear to me why the authors used both in-depth interviews and focus groups? And, how they did decide on what to use when. It seems that focus groups were sometimes done with only 2 participants? | **Response to reviewer**  FGD and IDI are both the choice for the qualitative data collection. IDI was specifically conducted with the clinic managers to avoid the power dynamic in FGD if they are present together with their staff. FGD was the choice for qualitative interviews with HCPs and patients respectively as the dynamics in the group might help to explore more findings. Participants can counter and support each other’s opinions leading to richer and more robust findings. However, for participants who were unable to participate in FGDs, IDIs were conducted to accommodate their schedules. We have now added this information in the Methods section.  **Page 6-7, line 93-100**  *In this study, in-depth individual interviews (IDI) and focus group discussions (FGD) were conducted. IDI was specifically conducted with the clinic managers to avoid the power dynamic in FGD if they are present together with their staff. FGD was the choice for qualitative interviews with HCPs and patients as the dynamics in the group might help to explore more findings. Participants can counter and support each other’s opinions leading to richer and more robust findings. However, for participants who were unable to participate in FGDs, IDIs were conducted to accommodate their schedules.* |
| 3. | Methods  Sampling a heterogeneous sample of participants is often a difficult task in qualitative research. Apparently, the contact person per clinic sampled the professional and patient interviewees. It may have the risk of bias, e.g. in selecting the less critical persons? | **Response to reviewer**  The clinic manager was asked to refer HCPs to participate in the qualitative interview and most of the time the diabetes team were referred to as they were the most relevant and fulfill the participant inclusion criteria. All the HCPs in the diabetes team were invited to participate hence there is a low likelihood that less critical persons were selected. As for Clinic C, which was without a diabetes team, indeed, the HCP participants were selected by the clinic manager.  Patient participants were actually referred to the researchers by medical officers who were practising in the clinic on the day the interviews were conducted with the clinic manager and the HCPs. Based on the patients’ characteristics in Table 2, it can be observed that patients of various education level were recruited and this suggests that it is unlikely that patients of certain characteristics were referred to participate in the interviews.  Nevertheless, we agree with the reviewer that there might be a risk of bias of participant selection and have included this in our study limitation. We have made participant recruitment process clearer in the manuscript and added a section on study limitation.  **Page 10-11, line 146-160**  **Data collection**  *All clinic managers agreed to participate and site meetings were set to know more about the clinic context as well as provide more information to the clinic managers. During the site meetings, WTT and YKL were brought around the sites and explained on the clinics’ running processes. Through this, the researchers were able to observe and understand each of the clinic contexts better. Then, the clinic managers were subsequently asked to refer researchers to staff who fulfilled the participant inclusion criteria for this study. In majority of the clinics, all the HCPs in the diabetes team were referred for participation, as they were the most relevant individuals except for Clinic C, which was without a diabetes team. The clinic manager of Clinic C selected the HCPs to participate. Appointments were then made with individuals who agree to participate to conduct the interviews. Patient participants were recruited by seeking help from the medical officers who were practising in the clinic on the day the interviews were conducted with the clinic managers and the HCPs. Participant response rate was 100% for both HCPs and patients. All the interviews were conducted in the clinics for the convenience of the participants.*  **Page 29, line 541-551**  **Study limitations**  *This study has a few limitations. First, in terms of setting and participant recruitment, Clinic C was included in this study despite its clinic manager being involved in the development of the insulin PDA. Hence, clinic manager C might have provided desirable responses.* ***Second, the clinic manager of Clinic C referred the HCP participants to participate in the interviews and patient participants were also referred to the researcher by medical officers who were practising in the clinics. There might have been risk of bias in the participant recruitment; clinic manager and medical officers selecting less critical persons to participate. However, all the participants in this study were asked to provide honest opinions and HCPs and patient were assured that their participation in this study would not affect their work and medical care.*** |
| 4. | Methods  It is not clear how the data collection was timed with respect to the implementation activities. Were the participants just in the beginning of the implementation period? Looking back after active implementation has ended? In the middle of the innovative change? (The first quote in theme 5 confused me. Is this a statement based on real experience. Or on expectation? | **Response to reviewer**  We apologise for the confusion that this study might have been thought as an implementation study. This study actually aims to prospectively identify factors influencing implementation of the insulin PDA in public community clinics in Malaysia. The findings of this study hope to inform future development of common and contextual implementation strategies to incorporate PDA use into standard clinical practice.  The first quote in Theme 5 is based on expectation.  We have now revised some of the texts in the quotes as well as throughout the manuscript to make it that clear that this is an exploratory study to identify factors influencing PDA implementation prior to active implementation.  **Page 6, line 73-77**  **Introduction**  *This study aims to* ***prospectively*** *identify factors influencing implementation of the insulin PDA in public community clinics in Malaysia. The findings of this study hope to inform* ***future*** *development of common and contextual implementation strategies to incorporate PDA use into standard clinical practice in all community clinics under the same organization for effective implementation of the insulin PDA.*  **Page 9, line 120-121**  **Methods**  **Study participants**  *Patients are included in this study to obtain a more comprehensive finding on* ***potential*** *factors that could influence the insulin PDA implementation is obtained.*  **Page 11, line 175-180**  **Data collection**  *The purpose of showing the video was to provide the participants with a clearer understanding of SDM and how the insulin PDA can be operationalize so they could think of the* ***potential*** *factors that would influence its implementation in their clinic settings. After viewing the video, the participants were asked to think about the* ***possible barriers and facilitators*** *and other ways the PDA can be implemented in the clinic.*  **Page 17, line 236-239**  **Results**  *Data analysis uncovered five themes that were* ***perceived as potential factors*** *that could influence the implementation of the insulin PDA in the five clinics and they were: time, patient load and lack of manpower; cost to print the PDA booklets; tailoring PDA use to patient profile; patient decisional role; and leadership and staff motivation.*  **Page 17, line 244-246**  **Theme 1: Time constraints, high patient load and lack of manpower**  *Time constraint* ***was raised as a major potential barrier*** *for implementation of PDA for all five clinics.*  **Page 19, line 286-291**  ***Theme 3: Tailoring PDA use to patient profile***  *Among the clinics from semi-urban areas where many patients were reported to be of lower education and socio-economic status (Clinic D and E), the clinic managers were concerned if the PDA is applicable to the patients in their setting due to patients’ ability to comprehend the information in the insulin PDA.* ***This was felt could be a prominent potential barrier for the insulin PDA implementation.***  **Page 19-20, line 305-308**  *While language barrier was also raised as a* ***potential barrier*** *for other clinics such as Clinic A and B, however, majority of their patients were educated and able to read. Most of their patients could speak either English or Malay fluently and furthermore there is multi-ethnic staff in the clinic who could help with translation.*  **Page 21, line 350-352**  **Theme 5: Leadership and staff motivation**  ***Another potential factor*** *influencing implementation of PDA is the clinic’s leadership and staff motivation.*  **Page 23-24, line 399-405**  **Discussions**  *This study uncovered the factors* ***that could influence*** *implementation of PDA in public health clinics under the same organization in Malaysia and revealed some important patterns. Factors such as time constraints, high patient load, lack of manpower and, cost that were raised as barriers to implementation in all the clinics were related to the resources provided by the Ministry of Health. On the other hand, some of the study clinics appeared to have distinct prominent factors that* ***might affect*** *their implementation of the insulin PDA such as clinics’ leadership and staff motivation and patient population and these are related to the individual context of the clinics.* |
| 5. | I was a bit shocked by the quote in theme 2. If a budget cut of 20% (!) is being set, implementation of Patient Decision Aids seems impossible…. Should this barrier be given more weight in the paper? | **Response to reviewer**  We have added more discussions on the impact of cost on the insulin PDA implementation.  **Page 25, Line 437-450**  **Discussion**  *Furthermore, PDA implementation may also be hampered by the financial constraints faced by all the clinics. Efforts are needed to obtain buy-in from the Ministry of Health to invest in PDA implementation so that specific allocation of the national healthcare funding can be provided. This may also help to sustain the PDA implementation. There is a need to show healthcare authority on the advantages of PDA in terms of improved patient care [2] as well reduced healthcare costs [45]. Studies have shown that when PDAs are used, fewer patients chose elective surgery compared to conservatory treatment and this helps to save the national healthcare expenditure [46, 47].* ***More local evidences are needed on the positive impact of the insulin PDA implementation to clinic services as well as patient health outcomes to obtain the clinics’ higher authorities buy-in to prioritise the insulin PDA implementation. An alternative to address the cost of PDA implementation is the use of web-based PDAs, which can be access freely by patients. However, this approach needs to be tested as a previous study on the acceptability of the insulin PDA have shown that HCPs and patients prefer booklet compared to electronic PDA (multimedia, video, and CDs) [29].*** |
| 6. | Theme 4, the 1st quote. “If SDM is looked upon as mainly letting the patient to make decision themselves…..” This is in my view a frequent occurring misconception of what SDM actually is about. It is NOT about the patient deciding for him or herself. Patients want support, want to share the decisional stress. While the doctor stays, of course, end-responsible for the decision. This may need a reflection in the discussion. Did the clinicians understand what SDM is really about? | **Response to reviewer**  *We thank the reviewer for pointing the misconception of SDM found among participants of this study. The short presentation which was given to the study participants prior to the interview might had not been sufficient to enable some of the HCPs to fully grasp the concept of SDM. SDM is generally a foreign concept in Malaysia in that SDM activities only began in 2010. While the Ministry of Health Malaysia and the Malaysian Medical Council has encourage doctors to perform SDM-related activities such as providing patients with accurate and timely information, discuss treatment limitation and risks, and collaborate with patient in selection of treatment since 1990s, patient involvement in healthcare decision making in Malaysia was still found to be lacking as there is no formal training and education, laws, regulations and health policies that supports SDM. SDM is not taught explicitly in the medical undergraduate and postgraduate curriculum. To implement SDM and PDA effectively, HCP training is warranted. Studies have reported the lack of training to be contributing factors to lack of engagement of patients in SDM and proper use of PDAs. The benefits of training are well evidenced and it should be a pre-requisite for SDM and PDA implementation. We have now included these information in the Discussion section:*  **Page 27-28, line 503-521**  *Apart from empowering patients, HCPs also need to be taught on the concept of SDM. Based on the qualitative narratives, it can be found that some HCPs might not had been able to fully grasp the concept of SDM (Theme 4, quote 1: “Shared decision making is mainly letting the patient to make the decision themselves…”) in the short presentation, which was given prior to the interview. HCPs need to understand their role in SDM in terms of providing quality information to patients and supporting patients in their deliberation of the treatment option [1] rather than placing the onus of health decision making to patients. SDM is generally a foreign concept in Malaysia in that SDM activities only began in 2010 [57]. While the Ministry of Health Malaysia and the Malaysian Medical Council has encourage doctors to perform SDM-related activities such as providing patients with accurate and timely information, discuss treatment limitation and risks, and collaborate with patient in selection of treatment since 1990s [57-59], patient involvement in healthcare decision making in Malaysia was still found to be lacking as there is no formal training and education, laws, regulations and health policies that supports SDM [65]. SDM is not taught explicitly in the medical undergraduate and postgraduate curriculum [57]. To implement SDM and PDA effectively, HCP training is warranted. Studies have reported the lack of training to be contributing factors to lack of engagement of patients in SDM and proper use of PDAs [66, 67]. The benefits of training are well evidenced [60, 61] and it should be a pre-requisite for SDM and PDA implementation [68].* |
|  |  |  |
| 7. | **Minor comments (Typos etc)** |  |
| 8. | Introduction: clarify -> clarifying | **Response to reviewer**  The grammatical error is corrected.  **Page 3, line 8**  *PDAs are tools that contain information on the health decision that needs to be made, information about the treatment options and outcomes, and by* ***clarifying*** *patients’ personal values. Its effectiveness has been well established in promoting patient-clinician communication and improving decision quality [2].* |
| 9. | Methods: Allow -> allowed | **Response to reviewer**  The grammatical error is corrected.  **Page 6, line 86-88**  *This study adopted a comparative case study design with a qualitative focus [32]. This study design was chosen because it* ***allowed*** *identification of similarities and differences of potential barriers and facilitators to implementing the insulin PDA across different implementation sites.* |
| 10. | Results  - Avoid acronyms like DE  - In theme 3 one quote is printed twice  - Theme 4 share decision making -> shared decision making  - …listen to doctor -> listen to the doctor  - Oh is only -> Oh it is only  - Complain here is slow -> ??? | **Response to reviewer**  We have removed the acronym “DE” and spelled it in full throughout the manuscript.  **Response to reviewer**  We have removed the quote duplicated in Theme 3.  **Response to reviewer**  We have corrected ‘share decision making’ to shared decision making.  **Page 21, line 330-333**  ***“Shared decision making*** *is mainly letting the patient to make the decision themselves, but our patients of the older generation rely on doctor to decide for them. So far, I’ve not come across any who said ‘Ok doctor I will do this, I will think over it and decide’”. - IDI 11_Clinic D_Clinic manager*  **Response to reviewer**  We have corrected ‘listen to doctor’ to ‘listen to the doctor’ and  ‘oh is only’ to ‘oh it is only’  **Page 21, line 345**  *“The patients here would only* ***listen to the doctor.*** *They don’t listen to nurses. Later when they come back for appointment, they will say ‘Oh it is only to see the nurses, never mind then’. That's the problem with educated patients laugh)”. - IDI 8_Clinic B_Diabetes educator*  **Response to reviewer**  We have added some texts to clarify the texts ‘Complain here is slow’  **Page 23, line 391**  *“The problem here is we are rushing to finish seeing patients as soon as possible. I received a complaint because I was slow. The culture here is that if you see a patient and if there is no problem you just give medicine and they go home. Patients also* ***complained (that the work process)*** *here is slow. Here you need to finish seeing one patient in five minutes (laughs). Since the patients are like that, the staff also had to follow the flow. Patients will scold if is taking too long. That’s why those who can read and understand and, has the patience they can take it home. For patients who are impatient, I don’t think they will appreciate this one (insulin PDA)”. - FGD 5_Clinic C_MO2* |
| 11. | Discussion  - Avoid acronyms like MOH  - Staff nurses has -> staff nurses have  - Doctors’ -> doctors  - They wanted the -> they wanted  - To patient -> to patients  - More involve -> more involved  - The intervention has been -> the interventions have been  - Involving of family -> involving family  - Support clinical champion -> support from a clinical champion  - Did increased -> did increase | **Response to reviewer**  We have removed the acronym ‘MOH’ and spell it in full ‘Ministry of Health’ throughout the manuscript and have corrected the pointed grammatical mistakes  **Page 24, line 429**  *In Malaysia, to be qualified as a diabetes educator, staff nurses* ***have*** *to complete*  **Page 25, line 432**  ***…doctors*** *in using the PDA with patients during consultation. Furthermore, PDA*  **Page 26, line 472**  ***they wanted someone*** *to aid them in PDA use in a straightforward, plain language and want*  **Page 19, line 289**  *Among the clinics from semi-urban areas where many patients were reported to be of lower education and socio-economic status (Clinic D and E), the clinic managers were concerned if the PDA would be applicable* ***to patients*** *in their setting due to patients’ ability to comprehend the information in the insulin PDA.*  **Page 27, line 494**  *study indicate that efforts to increase patients’ awareness on being more* ***involved*** *in their*  **Page 27, line 500**  *The* ***interventions have been*** *shown to be effective [55]…*  **Response to reviewer**  We have removed the texts involving of family members  **Page 28, line 526**  ***support from a clinical champion*** *is one of the key facilitators for effective PDA and SDM…*  **Response to reviewer**  Did increased -> did increase PDA : We have removed the texts from the manuscript. |
| **Reviewer #2** | | |
| **Abstract** | | |
| 12. | “PDA” is not fully spelled out at its first mention. | **Response to reviewer**  We thank the reviewer for highlighting this error. ‘PDA’ is now spelled out at first mention.  **Abstract**  **Page 2, line 4**  *Many* ***patient decision aids (PDAs)*** *are developed in academic settings by academic researchers.* |
| 13. | It is not clear how the first sentence of the abstract relates to the second sentence. | **Response to reviewer**  We have added a sentence to connect the first sentence of the abstract to the second sentence.  **Page 2, line 4-7**  *Many patient decision aids (PDAs) are developed in academic settings by academic researchers.* ***Academic settings are different from community clinics where the focus is on clinical work.*** *Thus, research on implementation in community settings will provide insights to effective implementation of PDA in real-world settings.* |
| 14. | A comparative case study design with a qualitative focus should be clarified better. More on this in the Methods sections. | **Response to reviewer**  We have now added the following texts to clarify the use of the comparative case study design in the Abstract as well as the Methods section.  **Page 2, line 12-14**  **Abstract**  **Methods**  *This study adopted a comparative case study design with a qualitative focus* ***to identify similarities and differences of the potential barriers and facilitators to implementing the insulin PDA across different implementation sites****.*  **Page 6, line 81-86**  **Methods**  **Study design**  *This study adopted a comparative case study design with a qualitative focus [32, 33].* ***This study design adopts similar approach as qualitative case study design whereby a phenomenon is studied in-depth by taking consideration of its context [32]. However, comparative case study design also allows understanding of similarities and differences across cases and is useful for understanding how context can influences the success of an intervention [32].***  **Page 13-14, line 211-218**  **Data analysis**  ***Align with the comparative case approach, which used various forms of data [32], field notes from the sites observations and interview reflections were also referred to help in understanding and interpretation of the data. Furthermore, data analysis were also performed sequentially starting from Clinic A to Clinic E that allowed WTT to fully immerse into the data for each clinic as well as making comparisons to other clinics as categories and emerged [32].*** *Subsequently, the themes and categories were compared across the five clinics and the similarities and differences of the findings were identified.* |
| 15. | Results: We start to get a sense that actually the analysis is basic, or it is summarized a bit simplistically. | **Response to reviewer**  We have reorganize the results and now provide more information by linking clinics’ profile to the themes emerged throughout the Results section.  **Page 2, line 20-32**  **Results**  *Five themes emerged which were time constraints, patient load and lack of manpower; cost to print the PDA booklets;* ***tailoring PDA use to patient profile****; patient decisional role; and leadership and staff motivation. Based on the interviews and drawing on observations and interview reflection notes, time constraints and patient load emerged as common prominent factors that cut across all the clinics, however the themes of tailoring PDA use to patient profile; patient decisional role; leadership and staff motivation contrasted due to the distinct challenges faced by specific clinics.* ***Among clinics from semi-urban areas with more patients from lower education and socio-economic status, patients’ ability to comprehend the insulin PDA and their tendency to rely on their doctors and family to make health decisions were felt to be a prominent barrier to the insulin PDA implementation. Staff motivation appeared to be stronger in most of the clinics where specific time was allocated to diabetes team to attend to diabetes patients. However, a lack of leadership might affect the insulin PDA implementation even though a diabetes team is present.*** |
| 16. | The numbered list is a bit awkward; it is ok to list them without numbering the themes. | **Response to reviewer**  We have now removed the numbers.  **Page 2, line 20-22**  **Results**  *Five themes emerged which were time constraints, patient load and lack of manpower; cost to print the PDA booklets; tailoring PDA use to patient profile; patient decisional role; and leadership and staff motivation.* |
| 17. | “HCPs” is not spelled out fully. Please review abbreviations, make sure they can be used in a PLOS ONE abstract, and I recommend using any abbreviations sparingly. | **Response to reviewer**  We have removed the ‘HCPs’ acronym in the abstract. HCP is now spelled out full.  **Methods**  **Page 2, line 15**  *Focus groups and individual interviews were conducted with 28* ***healthcare providers*** *and 15 patients from five public community health clinics under the Ministry of Health in Malaysia.* |
| 18. | “Successful implementation”: what is successful? What is implementation? After reading the article, I am not always clear on how these terms are used in this manuscript. | **Response to reviewer**  We have removed the term “successful” and replace it with the term ‘effective’. Implementation is defined in this study as the integration of the use of the insulin PDA in a clinic setting.  Effective implementation refers to the effective integration of the use of the insulin PDA in the clinic setting. We have now included the definition of implementation in the Introduction section.  **Page 2, line 35**  **Abstract**  **Conclusions**  *This study found that* ***effective implementation*** *of the insulin PDA across different public health clinics would depends on leadership and team commitment and the need to tailor PDA use to patient profile.*  **Page 5, line 57-61**  **Introduction**  *Nevertheless, the insulin PDA had been pilot tested at public university-based primary care clinics, public healthcare clinics and a private general practices, however, the findings published were mainly on the content and layout of the insulin PDA rather than on implementation [29].* ***In this study, implementation is defined as the integration of the insulin PDA use in a clinic setting.***  **Response to reviewer**  Replacement of the term “successful’ to ‘effective’ can be found at various sections of the manuscript.  **Page 22, line 372**  **Results**  *The Clinic B’s manager also voiced that* ***effective*** *implementation of the insulin PDA would depend on the diabetes MO in-charge of diabetes in the clinic.*  **Page 30, line 562-567**  **Conclusions**  *This study found that, despite having similar infrastructure and resources,* ***effective*** *implementation of the insulin PDA across different public health clinics would depend on leadership, team commitment and the need to tailor PDA use to patient profile. Policy makers should avoid a ‘one size fits all’ approach when implementing health interventions in the Malaysian primary care setting.* |
|  |  |  |
|  | **Introduction** |  |
| 19. | A fuller introduction would include discussion that addresses:  How big of a diabetes burden is there in Malaysia?  What is the proportion of those with diabetes who need insulin?  Has the PDA been piloted elsewhere?  How, where, and was it beneficial?  If it is shown to work elsewhere, but maybe not everywhere (such as at a community level), then the purpose of your research will be stronger. | **Response to reviewer**  We have now expanded the Introduction based the information suggested.  **Page 4-5, line 25-60**  **Introduction**  *Malaysia, located in South East Asia is an upper middle-income country with a multiethnic population comprised of three main ethnicities namely Malay (67.4%), Chinese (24.6%) and Indian (7.3%) [14]. Type 2 diabetes is a major health problem in Malaysia and its prevalence continues to rise from 13.4% in 2015 to 18.3% in 2019 [15]. Diabetes is a significant risk factor for cardiovascular disease, which is the main principle cause of death in Malaysia [16]. Based on the Malaysia National Diabetes Registry, 76.2% of patients with type 2 diabetes did not achieved the Malaysian glycaemic treatment target of HbA1c <6.5% and only 21.4 % were on insulin in 2012 [17]. The cost of care of diabetes and its complications was estimated at RM2.04 billion of which almost 70% was borne by the Malaysian government in 2011 [18]. Given the increasing prevalence of type 2 diabetes, more patients would require to consider insulin especially when insulin is now recommended for early treatment [19]. However, the decision to initiate insulin is difficult. Patient faced difficulties in making decision about insulin therapy due to numerous factors such as fear of pain and injection, misconceptions that insulin would caused kidney failure and blindness and the perception that the need for insulin indicates end stage of diabetes [20-23]. Hence, there is a need to support decision making for insulin use to control blood sugar and one way to do this is the use of PDA. PDA enables selection of treatment that is align with patients’ needs and preferences [2] and therefore this may increase their adherence to their chosen treatment and ultimately improve their glycaemic control. Early insulin initiation can prevent early onset of diabetes complications and this in turn may help the Malaysian government to control diabetes care costs.*  *In 2012, a locally developed insulin PDA (https://decisionaid.ohri.ca/AZsumm.php?ID=1558) [8, 26] has been developed to help patients make informed decision on insulin initiation, to promote patient centered care and SDM. The insulin PDA was developed based on the needs of healthcare policymakers, clinicians and patients in the local context, evidence from systematic review and decision support theories and framework [27]. It is available in the Malay, Chinese, Tamil and English language to cater to the multiethnic multilingual Malaysian population. The insulin PDA met 7/7 for decision aid criteria and 5/8 of reducing risk of bias criteria set by the International Patient Decision Aids Standards Collaboration, which assesses the quality and standards of a PDA [28]. However, formal implementation of the insulin PDA in routine practice has not been conducted. Nevertheless, the insulin PDA had been pilot tested at public university-based primary care clinics, public healthcare clinics and a private general practices, however, the findings published were mainly on the content and layout of the insulin PDA rather than on implementation [29].* |
| 20. | Also, there is no definition or clarification of what implementation means, what "successful" implementation looks like. Does it just mean implementing the PDA and utilizing it, or does it mean that it is utilized AND benefits patients? These are slightly different research questions, so "implementation" should be clarified. | **Response to reviewer**  Implementation is defined as the integration of the use of the insulin PDA in a clinic setting. We have now included this definition in the Introduction section.  **Page 5, line 57-61**  *Nevertheless, the insulin PDA had been pilot tested at public university-based primary care clinics, public healthcare clinics and a private general practices, however, the findings published were mainly on the content and layout of the insulin PDA rather than on implementation [29].* ***In this study, implementation is defined as the integration of the insulin PDA use in a clinic setting.***  **Response to reviewer**  We have removed the term “successful” and replace it with the term ‘effective’. Effective implementation refers to effective integration of the insulin PDA use in routine clinical setting. This had been clarified at the Introduction section.  **Page 3, line 11-15**  **Introduction**  ***It has been almost three decades since PDAs have emerged in the healthcare scene [2], however, its use in routine clinical setting is still lacking [3]. Effective implementation was hampered by*** *various factors such as time constraints, healthcare professionals’ attitude, patient characteristics, clinic capacity and processes of care and the healthcare environment [3-5].*  **Response to reviewer**  Replacement of the term “successful’ to ‘effective’ can be found at various sections of the manuscript.  **Page 22, line 372**  **Results**  *The Clinic B’s manager also voiced that* ***effective*** *implementation of the insulin PDA would depend on the diabetes MO in-charge of diabetes in the clinic.*  **Page 30, line**  **Conclusions**  *This study found that, despite having similar infrastructure and resources,* ***effective*** *implementation of the insulin PDA across different public health clinics would depend on leadership, team commitment and the need to tailor PDA use to patient profile. Policy makers should avoid a ‘one size fits all’ approach when implementing health interventions in the Malaysian primary care setting.* |
| 21. | Somewhere in the intro or in the methods, it is important to outline for the reader what the PDA is, who delivers it to the patients, and how. Because sometimes it sounds as if DE give the information, then it sounds like it's only the MO or doctors--it is unclear. If you are able to show how the different staff members in the clinics represented in table 1 participate (or don't participate) in PDA for insulin that would greatly contextualize your findings and make it easier for your reader. | **Response to reviewer**  More information about the insulin PDA is now included in the Introduction section.  **Page 4-5, line 47-61**  **Introduction**  *In 2012, a locally developed insulin PDA (https://decisionaid.ohri.ca/AZsumm.php?ID=1558) [8, 26] has been developed to help patients make informed decision on insulin initiation, to promote patient centered care and SDM. The insulin PDA was developed based on the needs of healthcare policymakers, clinicians and patients in the local context, evidence from systematic review and decision support theories and framework [27]. It is available in the Malay, Chinese, Tamil and English language to cater to the multiethnic multilingual Malaysian population. The insulin PDA met 7/7 for decision aid criteria and 5/8 of reducing risk of bias criteria set by the International Patient Decision Aids Standards Collaboration, which assesses the quality and standards of a PDA [28]. However, formal implementation of the insulin PDA in routine practice has not been conducted. Nevertheless, the insulin PDA had been pilot tested at public university-based primary care clinics, public healthcare clinics and a private general practices, however, the findings published were mainly on the content and layout of the insulin PDA rather than on implementation [29]. In this study, implementation is defined as the integration of the insulin PDA use in a clinic setting.*  **Response to reviewer**  We apologise for the confusion that this study might have been thought as an implementation study and we are looking back at after active implementation has ended. This study actually aims to prospectively identify factors influencing implementation of the insulin PDA in public community clinics in Malaysia prior to development of common and contextual implementation strategies to incorporate PDA use into standard clinical practice. Interviews with the participants helped to identify which HCP (i.e., doctors, diabetes educator, nurse, pharmacist) is appropriate to carry out specific tasks in future implementation of the insulin PDA.  We have now revised some of the texts in the quotes as well as throughout the manuscript to make it that clear that this is an exploratory study to identify factors influencing PDA implementation prior to active implementation.  **Page 6, line 73-77**  **Introduction**  *This study aims to* ***prospectively*** *identify factors influencing implementation of the insulin PDA in public community clinics in Malaysia. The findings of this study hope to inform* ***future*** *development of common and contextual implementation strategies to incorporate PDA use into standard clinical practice in all community clinics under the same organization for effective implementation of the insulin PDA.*  **Page 9, line 120-121**  **Methods**  **Study participants**  *Patients are included in this study to obtain a more comprehensive finding on* ***potential*** *factors that could influence the insulin PDA implementation is obtained.*  **Page 11, line 175-180**  **Data collection**  *The purpose of showing the video was to provide the participants with a clearer understanding of SDM and how the insulin PDA can be operationalize so they could think of the* ***potential*** *factors that would influence its implementation in their clinic settings. After viewing the video, the participants were asked to think about the* ***possible barriers and facilitators*** *and other ways the PDA can be implemented in the clinic.*  **Page 17, line 236-239**  **Results**  *Data analysis uncovered five themes that were* ***perceived as potential factors*** *that could influence the implementation of the insulin PDA in the five clinics and they were: time, patient load and lack of manpower; cost to print the PDA booklets; tailoring PDA use to patient profile; patient decisional role; and leadership and staff motivation.*  **Page 17, line 244-246**  **Theme 1: Time constraints, high patient load and lack of manpower**  *Time constraint* ***was raised as a major potential barrier*** *for implementation of PDA for all five clinics.*  **Page 19, line 286-291**  ***Theme 3: Tailoring PDA use to patient profile***  *Among the clinics from semi-urban areas where many patients were reported to be of lower education and socio-economic status (Clinic D and E), the clinic managers were concerned if the PDA is applicable to the patients in their setting due to patients’ ability to comprehend the information in the insulin PDA.* ***This was felt could be a prominent potential barrier for the insulin PDA implementation.***  **Page 19-20, line 305-308**  *While language barrier was also raised as a* ***potential barrier*** *for other clinics such as Clinic A and B, however, majority of their patients were educated and able to read. Most of their patients could speak either English or Malay fluently and furthermore there is multi-ethnic staff in the clinic who could help with translation.*  **Page 21, line 350-352**  **Theme 5: Leadership and staff motivation**  ***Another potential factor*** *influencing implementation of PDA is the clinic’s leadership and staff motivation.*  **Page 23-24, line 399-405**  **Discussions**  *This study uncovered the factors* ***that could influence*** *implementation of PDA in public health clinics under the same organization in Malaysia and revealed some important patterns. Factors such as time constraints, high patient load, lack of manpower and, cost that were raised as barriers to implementation in all the clinics were related to the resources provided by the Ministry of Health. On the other hand, some of the study clinics appeared to have distinct prominent factors that* ***might affect*** *their implementation of the insulin PDA such as clinics’ leadership and staff motivation and patient population and these are related to the individual context of the clinics.* |
| 22. | (http://dmit.um.edu.my/?modul=DMIT_PDA): it is unclear if this is the link that is meant. There is limited information available through this link. | **Response to reviewer**  We apologise for the non-working link provided. We now provide a new link.  **Page 4, line 48**  *In 2012, a locally developed insulin PDA* ***(https://decisionaid.ohri.ca/AZsumm.php?ID=1558)*** *[8, 26] has been developed to help patients make informed decision on insulin initiation, to promote patient centered care and SDM.* |
| 23. | “This study aims to explore the factors…”: I still fail to see why patients have been included at all as study participants. | **Response to reviewer**  Implementation science generally focuses on understanding and change HCPs’ behaviours as opposed to patient behaviours (Nilsen, Ståhl, Roback, & Cairney, 2013).  *Nilsen P, Ståhl C, Roback K, et al. Never the twain shall meet? - a comparison of implementation science and policy implementation research. Implementation Science. 2013;8(1):63.  In literature surrounding SDM and PDA implementation, patient's perspectives on implementation processes are often not reported eventhough they are the end-users of the innovation. Patient can contribute such as by informing from who and how they want the innovation to be delivered to them (Bhavnani & Fisher, 2010; Newsome et al., 2012; Crothers et al., 2016;).  *Bhavnani V, Fisher B. Patient factors in the implementation of decision aids in general practice: a qualitative study. *Health Expectations.* 2010;13(1):45-54.  * Newsome A, Sieber W, Smith M, et al. If you build it, will they come? A qualitative evaluation of the use of video-based decision aids in primary care. *Family medicine.* 2012;44(1):26-31.  We recognize the importance of engaging patients in the design and delivery of innovations as their characteristics, beliefs and attitudes can have a significant impact on implementation outcomes. Patients are included in this study to obtain a more comprehensive finding on potential factors that could influence the insulin PDA implementation is obtained. We have now provided the justification for including patients in the Methods section.  **Page 9, line 116-121**  *In literature surrounding SDM and PDA implementation, patient's perspectives on implementation processes are often not reported even though they are the end-users of the innovation. Patient can contribute such as by informing from who and how they want the innovation to be delivered to them [35-37]. Patients are included in this study to obtain a more comprehensive finding on potential factors that could influence the insulin PDA implementation is obtained.* |
|  |  |  |
|  | **Methods** |  |
| 24. | IDI, FGD: these are not fully described. Which method was used with which type of participant? Why were these methods chosen for the specific participants, context, and research question?  When you revise your methods, please follow the COREQ checklist: Allison Tong, Peter Sainsbury, Jonathan Craig, Consolidated criteria for reporting qualitative research (COREQ): a 32-item checklist for interviews and focus groups, International Journal for Quality in Health Care, Volume 19, Issue 6, December 2007, Pages 349–357, https://doi.org/10.1093/intqhc/mzm042 | **Response to reviewer**  IDI was specifically conducted with the clinic managers to avoid the power dynamic in FGD if they are present together with their staff. FGD was the choice for qualitative interviews with HCPs and patients as the dynamics in the group might help to explore more findings. Participants can counter and support each other’s opinions leading to richer and more robust findings. However, for participants who were unable to participate in FGDs, IDIs were conducted to accommodate their schedules. We have now added the information above in the Methods section.  **Page 6-7, line 94-100**  *In this study, in-depth individual interviews (IDI) and focus group discussions (FGD) were conducted. IDI was specifically conducted with the clinic managers to avoid the power dynamic in FGD if they are present together with their staff. FGD was the choice for qualitative interviews with HCPs and patients as the dynamics in the group might help to explore more findings. Participants can counter and support each other’s opinions leading to richer and more robust findings. However, for participants who were unable to participate in FGDs, IDIs were conducted to accommodate their schedules.*  **Response to reviewer**  We have also now reported our methods following the items in the COREQ checklist. Please refer to the COREQ checklist attached. |
| 25. | Table 1, “Patient profile (Ethnicity)”: Are the authors assuming that ethnicity determines language capacity? It seems from the results that language is more important then perhaps cultural elements that can be attributed to ethnicity. Would this be more important than ethnicity? | **Response to reviewer**  Malaysia has a multiethnic population, which comprised of three main ethnicities namely Malay, Chinese and Indian. In Malaysia ethnicity is proxy for the types of language spoken in an individual.  The national language in Malaysia is the Malay language, however, while Chinese and Indians can speak in the Malay language there are also some who may not have high Malay language proficiency. Chinese and Indians also have their own language namely Mandarin and Tamil. A doctor of Malay ethnicity may face the challenge in communicating with a Chinese and Indian patient should the patient unable to converse in Malay or in English. English is generally widely spoken in Malaysia however the proficiency varies from high to low as English is considered a second language. We have included the information above in Table 1, under *Notes.  **Page 8**  **Table 1: Characteristics of the public community clinics selected**  **Notes: In Malaysia, ethnicity is proxy for the types of language spoken in an individual. Malaysia has a multiethnic population, which comprised of three main ethnicities namely Malay, Chinese and Indian. The national language in Malaysia is the Malay language, however, while Chinese and Indians can speak in the Malay language there are also some who may not have high Malay language proficiency. Chinese and Indians also have their own language namely Mandarin and Tamil. English is generally widely spoken in Malaysia however the proficiency varies from high to low as English is generally considered as second language.* |
| 26. | Table 1 “illiterate”: Are these participants illiterate in their own languages? Illiterate in Malay? And how is this determined? | **Response to reviewer**  The researcher contacted 12 community clinics by email to ask about the characteristics of their clinics before finally selected five clinics selected to seek participation. The characteristics of the five clinics were based on the clinic managers’ responses to questions posed by the researcher on their clinic in terms of location, patient population profile (ethnicity, socio-economic status, education level) and presence of insulin support and manpower for diabetes management (Table 1). The term illiterate was used by one of the clinic manager to indicate patients’ education level. We have now removed the term ‘illiterate’ and changed it to low educational level.  **Page 8**  Table 1: Characteristics of the public community clinics selected |
| 27. | Table 1 “Manpower”: One may wonder if manpower also relates to number of patients seen at a clinic. Should this also be mentioned as a defining characteristic of the different clinics? | **Response to reviewer**  Manpower refers to staffing and not related to the number of patients seen at a clinic. We have now changed the term ‘manpower’ to **‘manpower (staffing)’** for a clearer description.  **Page 8**  Table 1: Characteristics of the public community clinics selected |
| 28. | Throughout the methods there is mention of FGD, then there is seemingly a description of only interviews in this section. Then we come to the results tables and we find that indeed, focus groups were performed.  Who were included in interviews and who were included in focus groups and how were the focus group guides created? | **Response to reviewer**  In-depth interviews (IDIs) refers to individual interview. Therefore, in this study, two types of interviews were conducted; individual interview and focus group. IDI was specifically conducted with the clinic managers to avoid the power dynamic in FGD if they are present together with their staff. FGD was the choice for qualitative interviews with HCPs and patients respectively as the dynamics in the group might help to explore more findings. Participants can counter and support each other’s opinions leading to richer and more robust findings. However, for participants who were unable to participate in FGDs, IDIs were conducted to accommodate their schedules.  We have now changed the term “in-depth interview” to “in-depth individual interview” to make it clearer that it is a one-to-one interview as well as added the information on who were involved in the IDI and FGD in the Methods section.  **Page 6-7, line 93-100**  *In this study, in-depth individual interviews (IDI) and focus group discussions (FGD) were conducted. IDI was specifically conducted with the clinic managers to avoid the power dynamic in FGD if they are present together with their staff. FGD was the choice for qualitative interviews with HCPs and patients as the dynamics in the group might help to explore more findings. Participants can counter and support each other’s opinions leading to richer and more robust findings. However, for participants who were unable to participate in FGDs, IDIs were conducted to accommodate their schedules.*  **Response to reviewer**  The interview guide in this study was developed based on the Theoretical Domains Framework, literature review and discussions among researchers and then adapted to according to the types of participants; healthcare policy maker, healthcare providers and patients. We have included this information under the Methods section.  **Page 9-10, line 124-135**  *A semi-structured interview guide was utilized for this study. The development of the interview guides were informed by the Theoretical Domains Framework (TDF) [38], literature review and discussions among researchers. The TDF was selected because implementation or adoption of the insulin PDA is primarily dependent on behavior change of the healthcare providers and patients. The TDF consists of 14 theoretical domains synthesized from 33 behaviour change theories and can guide in development of interventions targeting at behavior change. Besides taking account of rational and cognitive process, the TDF also covers emotional and organizational factors such as working environment and resources [39] that can help to elicit barriers and facilitators to implementation or adoption of the insulin PDA. Questions and prompts were created according to the domains in the TDF and adapted according to healthcare policymaker, HCP and patient participants (Appendix 1).* |
| 29. | It is unclear why showing the video on appropriate PDA use would have been part of the design? This also may bias the responses because you showed a way that PDA could be used? | **Response to reviewer**  SDM and PDA is a foreign concept in Malaysia. The participants had not been exposed to or had no experience in using the insulin PDA. They were only introduced to and familiarised with SDM and the insulin PDA prior to the interviews. There is a possibility that the insulin PDA may be likened to another educational material hence some issues pertaining to the implementation processes for SDM and the insulin PDA might have been oversight. Therefore, the purpose of showing the video to the participants prior to the interviews is to provide them with a clearer understanding of SDM and how the insulin PDA can be operationalize so they could think of the factors that would influence its implementation in their clinic settings. However, the participants have also been informed that the video was just showing one way the PDA can be implemented and they were asked to think about the possible barriers and facilitators and other ways the PDA can be implemented in the clinic. We have included this information in the Methods section.  **Page 11, line 169-180**  *Prior to the interviews, the researchers gave the study information sheet and explained to the participants the purpose of the study, information on the insulin PDA, the concept of SDM and the various PDA modalities (booklet, tablet, website) available before informed consent was obtained from the participants. Given that SDM and PDA is a foreign concept in Malaysia, a video, which demonstrates how the insulin PDA can be used during a consultation, was also shown to all the participants to give them a clear picture on one of the ways the insulin PDA can be implemented in the clinic. The purpose of showing the video was to provide the participants with a clearer understanding of SDM and how the insulin PDA can be operationalize so they could think of the potential factors that would influence its implementation in their clinic settings. After viewing the video, the participants were asked to think about the possible barriers and facilitators and other ways the PDA can be implemented in the clinic.*  **Response to reviewer**  We also acknowledge that HCPs might not had been able to fully grasp the concept of SDM and the insulin PDA despite the short presentation given prior to interviews, hence we have now incorporated this as a study limitation.  **Page 29, line 551-555**  **Study limitations**  *Third, the participants had not been exposed to or had no experience in using the insulin PDA. They were only introduced and familiarised with SDM and the insulin PDA prior to the interviews. There is a possibility that the insulin PDA may be likened to another educational material hence some issues pertaining to the implementation processes for SDM and the insulin PDA might have been oversight.* |
| 30. | Because it's not clear with whom IDI or FGD were conducted, these methods are not clear. Did you show a video to all participants, including diabetic patients? | **Response to reviewer**  The IDIs were conducted with clinic managers while both IDIs and FGDs were conducted with HCPs and patients. The details on who the IDIs and FGDs were conducted with is detailed in Appendix 2. The video was shown to all the participants including patient participants before the IDIs and FGDs. This information is now included in the Methods section.  **Page 11, line 172-175**  *Given that SDM and PDA is a foreign concept in Malaysia,* ***a video, which demonstrates how the insulin PDA can be used during a consultation, was also shown to all the participants*** *to give them a clear picture on one of the ways the insulin PDA can be implemented in the clinic.* |
| 31. | Data analysis: The tools used for the interview guides are made to sound very strong. However, it seems that their potential for a deeper analysis was limited. The analysis could have been better guided by using the theoretical domains mentioned in the methods as a conceptual framework to perform your thematic analysis. | **Response to reviewer**  The interview guide was developed based on the Theoretical Domains Framework, however, we did not analyse our data according to the domains in the framework for two reasons:   - Firstly, we employed the TDF as the conceptual framework to develop the topic guide. We believe that using the TDF to conduct framework analysis may constrain the data to domains of the TDF; on the other hand, using a thematic approach would allow us to work through domains, which might not fit exactly with TDF domains. We therefore chose a thematic analysis approach to allow findings to be generated based on themes grounded in the data. - Secondly, a more open coding would allow us to explore patient-related domains as the TDF is mainly developed for identification of factors influencing healthcare professionals’ behaviours related to implementation of evidence-based interventions (Atkins et al., 2017*), with few studies that looked into patients’ perspective on implementation issues.   *Atkins L, Francis J, Islam R, O'Connor D, Patey A, Ivers N, Foy R, Duncan EM, Colquhoun H, Grimshaw JM, Lawton R, Michie S. A guide to using the Theoretical Domains Framework of behaviour change to investigate implementation problems. Implement Sci. 2017 Jun 21;12(1):77. doi: 10.1186/s13012-017-0605-9. |
| 32. | In addition, if you use a case studies approach to your qualitative analysis, I recommend reviewing qualitative analysis texts: Creswell JW. Qualitative Inquiry & Research Design: Choosing Among Five Approaches. London: SAGE; 2007.  Some good instruction on performing qualitative analysis: Miles MB, Huberman AM, Saldaña J. Qualitative data analysis: a methods sourcebook. 4 ed. Los Angeles: Sage; 2020. See Chs 4, 6, 9. | **Response to reviewer**  We thank the reviewer for the suggestions. We have now included more description on how the qualitative data analysis was performed. We referred to Glaser (1978)* in our qualitative data analysis and Mills et al., (2010) for case study approach.  *B.G. Glaser. Theoretical sensitivity: Advances in the methodology of grounded theory. (Vol 2). Mill Valley, CA: Sociology Press 1978.  *A. Mills, G. Durepos, E. Wiebe. Comparative Case Study. In: Mills A, Durepos G, Wiebe E, editors. Encyclopedia of Case Study Research. Thousand Oaks, California: SAGE; 2010.  We have now included more description on the data analysis.  **Page 12, line 193-198**  *Initially, transcripts were read line by line and codes (short phrases label) were assigned to specific data sections that represented their significance (open coding). Then, codes that were developed were reviewed and group together to form categories (axial coding). Related categories were then reviewed and overarching themes were applied that reflected the meaning of the data. Once the coding framework was developed, it was then used to code data from other transcripts (selective coding) [40].*  **Page 13-14, line 202-223**  *The data analysis was performed case by case for each clinic at a time in order to gain in-depth understanding on the contextual factors that influence implementation of the insulin PDA at the specific setting. Initially, the researchers WTT and YKL read the transcripts in detail from Clinic A and coding was performed independently. Categories and themes emerged were discussed and finalized when discrepancies were resolved. The finalized themes and categories for Clinic A were later used as a coding framework for data analysis for the rest of the clinics. Any new codes and categories that emerged were added to the coding framework while those that were not relevant were removed. WTT coded all the transcripts for all the clinics while YK coded for Clinic B and C, PYL coded for Clinic C and clinic D and CJN coded for Clinic E, independently. Align with the comparative case approach, which used various forms of data [32], field notes from the sites observations and interview reflections were also referred to help in understanding and interpretation of the data. Furthermore, data analysis were also performed sequentially starting from Clinic A to Clinic E that allowed WTT to fully immerse into the data for each clinic as well as making comparisons to other clinics as categories and emerged [32]. Subsequently, the themes and categories were compared across the five clinics and the similarities and differences of the findings were identified. The findings comparison across the clinics was indeed challenging, time consuming and exhausting as the researchers had to go through the transcripts, field notes, interview reflections for both HCPs and patients data from each clinics back and forth in order to understand the data thoroughly. However, by going back to the data again and again, soon the patterns of commonalities and differences in the barriers and facilitators across the clinics became clear to the researchers.* |
|  | **Results** |  |
| 33. | Table 2: I don't find this table helpful. It needs to be summarized better by interview type and study site. You can then add a range of values for different characteristics--for example for the FGD. | **Response to reviewer**  We have now summarized Table 3 as well as provide detail information of individual participant in each clinic in Appendix 2.  Page 15  Table 3: Participants’ socio-demographic information by clinic  Appendix 2: Detailed information of individual participant in each clinic |
| 34. | This table also makes me wonder more about the methods:  what determined which participants would be used in interviews or focus groups?  It is clear that you had interviews with patients.  But sometimes you mixed members of teams in a focus group at a given clinic?  Would there be different bias inherent in the data collected from a discussion with professionals with different training, expertise, experience, hierarchy, power dynamics? Would this benefit/hurt the quality of the data collected? How?  Why not conduct FGD with patients? It seems that this may be a more efficient way to elicit quality data from a patient perspective, and also enhance your ability to reach far more patients. These types of issues should be considered more fully in your Methods section. Again, refer to the COREQ checklist recommended above. | **Response to reviewer**  FGD and IDI are both the choice for the qualitative data collection. IDI was specifically conducted with the clinic managers to avoid the power dynamic in FGD if they are present together with their staff.  FGD was the choice for qualitative interviews with HCPs and patients respectively as the dynamics in the group might help to explore more findings. PDA implementation may involve more than one HCP and by getting various HCPs together in a FGD enabled various stakeholders to provide their opinions on the barriers and facilitators to the PDA implementation with respect of their roles and work processes in the clinic. The various HCP can counter or support each other’s opinions leading to richer and more robust findings. During FGDs with different HCPs, we did not sense any power dynamics that inhibited any participants from expressing their views. Another factor that determined the composition of HCP in the FGD and the choice of FGD or IDI is the convenience of the participants. Due to high workload and time constraints, some HCP and patients were unable to participate in FGDs, thus IDIs were conducted to accommodate their schedules. We have added information above to provide a clearer description to readers on the choice of IDI and FGD.  **Methods**  **Page 6-7, line 93-100**  *In this study, in-depth individual interviews (IDI) and focus group discussions (FGD) were conducted. IDI was specifically conducted with the clinic managers to avoid the power dynamic in FGD if they are present together with their staff. FGD was the choice for qualitative interviews with HCPs and patients as the dynamics in the group might help to explore more findings. Participants can counter and support each other’s opinions leading to richer and more robust findings. However, for participants who were unable to participate in FGDs, IDIs were conducted to accommodate their schedules.* |
| 35. | Paragraph after Table 2 “implementation”: I get the sense that this is not the appropriate term or technically what is being studied here. What does implementation mean? Since this is central to your study, it should be defined and discussed much earlier in your manuscript. | **Response to reviewer**  Implementation is defined as the integration of the use of the insulin PDA in a clinic setting. We have now included this definition in the Introduction section.  **Page 5, line 57-61**  *Nevertheless, the insulin PDA had been pilot tested at public university-based primary care clinics, public healthcare clinics and a private general practices, however, the findings published were mainly on the content and layout of the insulin PDA rather than on implementation [29].* ***In this study, implementation is defined as the integration of the insulin PDA use in a clinic setting.*** |
| 36. | 12. FGD 3_Clinic B_Patient 1 quote, “I don’t think our doctors don’t have time to explain”: Mistranslated? I believe it's meant that the doctors DON'T have time to explain. | **Response to reviewer**  We thank the reviewer for pointing out this error. We have made correction to the quote.  **Page 17, line 254**  *“I don’t think our* ***doctors have the time to explain*** *to patients. The amount of patients is almost 1000 patients, do you think doctor will open the book (PDA)?” - FGD 3_ Clinic B_Patient 1* |
| 37. | 13. Theme 2, IDI5_Healthcare policymaker_Clinic B, “We don’t have money to print”: Implicit here is rationing and the need to prioritize at a local community level. However, it would be more interesting to hear why PDAs were not prioritized—and how might this contrast to other forms of patient education? I think if it is low priority it may give a deeper sense of why implementation may not be widespread or differ from clinic to clinic. | **Response to reviewer**  SDM and PDA is generally a foreign concept in Malaysia hence more efforts are still needed to promote their concepts and use. At the point of the study, the clinics have yet to experience utilizing the insulin PDA. This study is an exploratory study to identify factors influencing PDA implementation prior to active implementation. More local evidences are needed on the positive impact of the insulin PDA implementation to clinic services as well as patient health outcomes to obtain the clinics’ higher authorities buy-in to prioritise the insulin PDA implementation. We have discussed the point above in the Discussion section.  **Page 25, line 437-450**  **Discussion**  *Furthermore, PDA implementation may also be hampered by the financial constraints faced by all the clinics. Efforts are needed to obtain buy-in from the Ministry of Health to invest in PDA implementation so that specific allocation of the national healthcare funding can be provided. This may also help to sustain the PDA implementation. There is a need to show healthcare authority on the advantages of PDA in terms of improved patient care [2] as well reduced healthcare costs [45]. Studies have shown that when PDAs are used, fewer patients chose elective surgery compared to conservatory treatment and this helps to save the national healthcare expenditure [46, 47]. More local evidences are needed on the positive impact of the insulin PDA implementation to clinic services as well as patient health outcomes to obtain the clinics’ higher authorities buy-in to prioritise the insulin PDA implementation. An alternative to address the cost of PDA implementation is the use of web-based PDAs, which can be access freely by patients. However, this approach needs to be tested as a previous study on the acceptability of the insulin PDA have shown that HCPs and patients prefer booklet compared to electronic PDA (multimedia, video, and CDs) [29].* |
| 38. | Theme 3, Patient profile: This section should be re-thought. If this is about SDM, this strikes me that the main theme emerging here is not the fault of the patients, but the fault of the PDA not being tailored to specific patient needs.  I note that no patient accounts of how they are limited in understanding the materials, if that is what is being asserted, such evidence from patient interviews would support this. But even if the patients are limited, the crux of the problem lies in the materials being inappropriate, not particularly a problem with the patients themselves.  If we follow the authors’ logic, one would conclude that the patient needs to be educated in the national languages or have to rise (or be raised) in their SES to get over their limitations to make PDA implementation possible. This can’t be what the authors intend to present and it can’t be what health policy makers would benefit from learning.  A more thoughtful analysis would instead consider that the materials not being tailored to the particularly diverse Malaysian population is really the problem. | **Response to reviewer**  The insulin PDA in this study was developed based on patients’ needs and met the readability criteria set by the International Patient Decision Aids Standards Collaboration. The pilot study on the acceptability of the insulin PDA found that patients viewed the content of the PDA to be simple and clear (Lee et al., 2016)*.  *Lee, P. Y., Khoo, E. M., Low, W. Y., Lee, Y. K., Abdullah, K. L., Azmi, S. A., & Ng, C. J. (2016). Mismatch between health-care professionals' and patients' views on a diabetes patient decision aid: a qualitative study. *Health Expect,* 19No., 427-436  The insulin PDA has also been adapted to four different languages to cater to the Malaysian multiethnic population. Nevertheless, patient low literacy level and language barrier was still brought up to be potential barriers to the insulin PDA implementation in this current study. Implementation of PDAs is not only about getting patients to be able to use them but involves implementation of a true dialogue and sharing of decision making attributes, values and preferences between doctor and patient. The issue with language barrier in relation to PDA implementation refers to the communication between HCP and patient when discussing information related to the insulin PDA.  We have now provided a patient’s account on the possibility that some patients may not be able to understand the insulin PDA.  We have also discussed the need to tailor information to patients as well as the potential use of videos and having someone to aid patients in PDA use in a straightforward, plain language to help address literacy issue. We have now changed the theme “patient profile’ to ‘tailoring PDA use to patient profile’.  **Page 19, line 286**  Theme 3: Tailoring PDA use to patient profile  **Page 19, line 298-299**  *“Some patients are not very well educated. So they may not understand what they read and what this is it all about.” IDI 7_Patient_Community clinic A*  **Page 26-27, line 466-482**  **Discussions**  *Very few PDAs developed took account of low health literacy and there are only few studies on interventions designed to mitigate low health literacy in PDA use [48]. The use of video images in PDAs have been shown to help reduce decision uncertainties among patients with lower objective health literacy [49]. It has also been suggested that when implementing PDA, HCPs should deliver PDAs to patients in the context of clinical care [48, 50]. In a study among low income racially diverse patients, the participants have voiced that they wanted someone to aid them in PDA use in a straightforward, plain language and wanted the information delivery to be tailored to individual patients in terms of the levels of information [37]. Adopting these strategies may help to facilitate patients’ attention and understanding of the information in the PDAs. Notwithstanding, barriers such as old age, ethnicity and education level were often thought as non-modifiable hence preventing PDA from being used, however, these barriers could be linked to attitudes and prejudices that can be addressed as well as provision of alternative strategies. For example, patients who are of old age who believe that they should not challenge clinicians [51] may be addressed by patient activation strategies [52]. Patient who are elderly with dementia can be addressed by providing different design, format and delivery style of PDAs [53].* |
| 39. | “The patient profile in the clinics pose distinct barriers”: I struggle that in a study trying to improve SDM, we still harp on the "patients" being portrayed as something of an obstacle. This reads a bit paternalistic, condescending. | **Response to reviewer**  We have now removed the sentence and revised the texts.  **Page 19, line 287-291**  *Among the clinics from semi-urban areas where many patients* *were reported to be of lower education and socio-economic status (Clinic D and E), the clinic managers were concerned if the PDA would be applicable to patients in their setting due to patients’ ability to comprehend the information in the insulin PDA. This was felt could be a prominent potential barrier for the insulin PDA implementation.* |
| 40. | IDI 16_Healthcare policy maker_Clinic E: I find two concepts in this quote, but I find one to be particularly problematic.  The first part casts doubt on the suitability of the materials, but the second part has a bit of a condescending view towards the patients themselves. Perhaps if more policy makers felt this way, this becomes a barrier to implementation--the prejudice of those in management or higher positions that believe some inherent limitations of the patients are what prevent the PDA from being used. This strikes me as a prejudice among higher level officials that is problematic, less so than the patients' themselves. However, it could be bolstered by a patient perspective that cannot understand the material, but again, the theme here would be the appropriateness of the materials for PDA as they relate to whether or not they are utilized at a given clinic. | **Response to reviewer**  We have changed the theme from “patient profile’ to ‘tailoring PDA use to patient profile’.  **Page 19, line 286**  Theme 3: Tailoring PDA use to patient profile  **Response to reviewer**  We have provided a patient’s account on the possibility that some patients might not be able to understand the insulin PDA.  **Page 19, line 298-299**  *“Some patients are not very well educated. So they may not understand what they read and what this is it all about.” IDI 7_ Community clinic A_Patient*  **Response to reviewer**  We have now included discussions on the perception that patient-related barriers such as old age, ethnicity and education level were often thought as non-modifiable hence preventing PDA from being used, however, these barriers could be linked to attitudes and prejudices that can be addressed as well as provision of alternative strategies.  **Page 26-27, line 476-482**  *Notwithstanding, barriers such as old age, ethnicity and education level were often thought as non-modifiable hence preventing PDA from being used, however, these barriers could be linked to attitudes and prejudices that can be addressed as well as provision of alternative strategies. For example, patients who are of old age who believe that they should not challenge clinicians [50] may be addressed by patient activation strategies [51]. Patient who are elderly with dementia can be addressed by providing different design, format and delivery style of PDAs [52].* |
| 41. | Theme 4, “The credibility of the PDA”: I point out comments above that this is a good example where a clear explanation of how and by whom the PDA information is given to the patient would be helpful to the reader. The PDA as you have put forth is provided by the doctor. So I am unclear what the nurses have to do with this? | **Response to reviewer**  We apologise for the confusion that this study might have been thought as an implementation study. This study actually aims to prospectively identify factors influencing implementation of the insulin PDA in public community clinics in Malaysia. Interviews with the participants identified which HCP is appropriate to carry out specific tasks in future implementation of the insulin PDA. It has been noted by the participants that patients may not likely to want to return to the clinic to see the nurses as compared to doctors for the follow-up on the insulin PDA. |
| 42. | 18. Theme 5, “The emphasis on diabetes management in the clinic claimed to be moderate”: What does moderate mean? | **Response to reviewer**  After reviewing the texts in the paragraph, we found that the sentence “The emphasis on diabetes management in the clinic claimed to be moderate” is not necessary and we have now removed the sentence.  **Page 23, line 380-386**  *At Clinic C, which was without a diabetes team, the clinic manager noted that diabetes leadership is low in the clinic and this might lead to ineffective implementation of the insulin PDA. ~~The emphasis on diabetes management in the clinic was claimed to be moderate.~~ The diabetes educator was reported to have little enthusiasm while the doctor in-charge of diabetes is relatively new in the diabetes team having just been trained for one month (at the time of the interview) to coordinate diabetes management in the clinic. Furthermore, HCPs were also reported to try to get their work done in a rush due to patients’ who are in a hurry to leave the clinic.* |
| 43. | Overall, this is a very basic qualitative analysis. The way it reads is a bit unclear what exactly the emergent themes were, and I am unsure about their validity in supporting the authors’ conclusions. It seems that the results focus on specific, mundane particulars about the clinics themselves without clear illustration of something more comprehensive picture that a strong thematic analysis would provide. | **Response to reviewer**  We have reflected on our findings and have improved the depth of the analysis. We have now reorganize the results and provide more information by linking clinics’ profile to the themes emerged.  **Page 19, line 286-291**  **Theme 3: Tailoring PDA use to patient profile**  *Among the clinics from semi-urban areas where many patients were reported to be of lower education and socio-economic status (Clinic D and E), the clinic managers were concerned if the PDA would be applicable to patients in their setting due to patients’ ability to comprehend the information in the insulin PDA. This was felt could be a prominent potential barrier for the insulin PDA implementation.*  **Page 20-21, line 323-333**  **Theme 4: Patient decisional role**  *Patients desire for involvement in SDM with doctors vary between clinics where there were more patients who were educated and from the middle-income group (Clinic A and B) compared to clinics where many patients were of lower educational level (Clinic D and E). It was noted that majority of the patients in the latter clinics rely on their doctors and family members to make health decisions for them, especially the older patients, hence the PDA may not be suitable to be used with such patients.*  *“Shared decision making is mainly letting the patient to make the decision themselves, but our patients of the older generation rely on doctor to decide for them. So far, I’ve not come across any who said ‘Ok doctor I will do this, I will think over it and decide’”. - IDI 11_Clinic D_Clinic manager*  *At clinics where patients were generally of high education level (Clinic A and B), patients appeared more empowered and would be more likely to be involved in decision making. The credibility of the PDA would be an important criterion that the patients would consider as it was noted that some patients would actually look at the rank of the doctors when seeking consultations. In addition, patients may not be willing to return to the clinic to meet the nurses as compared to doctors should the insulin PDA is implemented.*  **Page 21-22, line 350-357**  **Theme 5: Leadership and staff motivation**  *Another potential factor influencing implementation of PDA is the clinic’s leadership and staff motivation.* ***Staff motivation appeared to be stronger in most of the clinics where specific time was allocated to diabetes team to attend to diabetes patients (Clinic A, D and E).*** *Clinic A and D noted that the implementation of the insulin PDA can be carried without the need for directives from higher authority once they are educated on the advantages of the intervention. Staff training, empowerment and acknowledgement will motivate the staff to implement the PDA at their own will.*  **Page 22-23, line 367-386**  *Nevertheless, having a diabetes team was not always a guarantee of PDA implementation as Clinic B demonstrated that leadership support was necessary. Drawing on observation and interview reflection notes, the current leadership appeared to be lacking in Clinic B and this might affect the insulin PDA implementation even though a diabetes team is present. Diabetes may not be Clinic B’s health priority as the clinic manager’s key-focus is on another health area. The Clinic B’s manager also voiced that effective implementation of the insulin PDA would depend on the diabetes MO in-charge of diabetes in the clinic. However, during the researcher’s (WTT) interview with the diabetes MO in-charge, the individual demonstrated a lack of interest with the insulin PDA and was also in a hurry to end the interview session. The HCPs in Clinic B have also reported to not like to spend time talking to patients and the insulin PDA may be used as a substitute for consultation. Furthermore, staff nurses may not have a sense of responsibility of using the insulin PDA as they are on rotation and may perceived that it is the diabetes educators’ responsibility.*  *At Clinic C, which was without a diabetes team, the clinic manager noted that diabetes leadership is low in the clinic and this might lead to ineffective implementation of the insulin PDA. The diabetes educator was reported to have little enthusiasm while the doctor in-charge of diabetes is relatively new in the diabetes team having just been trained for one month (at the time of the interview) to coordinate diabetes management in the clinic. Furthermore, HCPs were also reported to try to get their work done in a rush due to patients’ who are in a hurry to leave the clinic.* |
|  |  |  |
|  | **Discussions** |  |
| 44. | “There is a need for implementation strategies to be tailored to address the contextual needs at each implementation site”: Yes, but unfortunately, I think your analysis was very limited if this was the reason for your study. You would have been better placed to take a bottom-up approach to your study design if this statement reflects the authors’ purpose. But instead, your study design took a top down approach, which the authors themselves argue is not appropriate in the introduction. Your data emphasizes policy makers, medical officers, other health staff, and lastly the patients. | **Response to reviewer**  While it is desirable to engage patients in the development implementation intervention, however, implementation science generally focuses on understanding and change HCPs’ behaviours as opposed to patient behaviours (Nilsen et al., 2013).  *Nilsen P, Ståhl C, Roback K, et al. Never the twain shall meet? - a comparison of implementation science and policy implementation research. Implementation Science. 2013;8(1):63.  Nevertheless, we recognize the importance of engaging patients in the design and delivery of innovations as their characteristics, beliefs and attitudes can have a significant impact on implementation outcomes. Therefore, in this current, we tried to obtain a comprehensive view on factors influencing PDA implementation by including healthcare policy makers, HCPs and patients. |
| 45. | “Efforts are needed to obtain buy-in from the ministry to invest in PDA…”: Yes, and this is why I recommend that in the introduction you state the burden of this particular problem. If you want policy makers to utilize your paper, cost of care information should also be provided if there is already existing data within Malaysia. | **Response to reviewer**  We thank the reviewer for the suggestion. We have now provided information on the burden of type 2 diabetes as well as the cost of care of diabetes and its complications in Malaysia.  **Page 4-5, line 25-50**  **Introduction**  *Malaysia, located in South East Asia is an upper middle-income country with a multiethnic population comprised of three main ethnicities namely Malay (67.4%), Chinese (24.6%) and Indian (7.3%) [14]. Type 2 diabetes is a major health problem in Malaysia and its prevalence continues to rise from 13.4% in 2015 to 18.3% in 2019 [15]. Diabetes is a significant risk factor for cardiovascular disease, which is the main principle cause of death in Malaysia [16]. Based on the Malaysia National Diabetes Registry, 76.2% of patients with type 2 diabetes did not achieved the Malaysian glycaemic treatment target of HbA1c <6.5% and only 21.4 % were on insulin in 2012 [17]. The cost of care of diabetes and its complications was estimated at RM2.04 billion of which almost 70% was borne by the Malaysian government in 2011 [18]. Given the increasing prevalence of type 2 diabetes, more patients would require to consider insulin especially when insulin is now recommended for early treatment [19]. However, the decision to initiate insulin is difficult. Patient faced difficulties in making decision about insulin therapy due to numerous factors such as fear of pain and injection, misconceptions that insulin would caused kidney failure and blindness and the perception that the need for insulin indicates end stage of diabetes [20-23]. Hence, there is a need to support decision making for insulin use to control blood sugar and one way to do this is the use of PDA. PDA enables selection of treatment that is align with patients’ needs and preferences [2] and therefore this may increase their adherence to their chosen treatment and ultimately improve their glycaemic control. Early insulin initiation can prevent early onset of diabetes complications and this in turn may help the Malaysian government to control diabetes care costs.*  *In 2012, a locally developed insulin PDA (https://decisionaid.ohri.ca/AZsumm.php?ID=1558) [8, 26] has been developed to help patients make informed decision on insulin initiation, to promote patient centered care and SDM.* |
| 46. | “There is a need to show healthcare authority…”: PDAs may be important, in general, but you haven't shown how they're important for diabetes (the literature you cite seldom discusses PDA forT2DM, important in the Malaysian context, of specifically how PDAs are useful to support insulin treatment in diabetes. If this is the assertion you want to make in the discussion, I would place the argument for it in the introduction. | **Response to reviewer**  We have now included information on how the insulin PDA can help to support decision making in diabetes treatment as suggested in the Introduction section.  **Page 4-5, line 25-50**  **Introduction**  *Malaysia, located in South East Asia is an upper middle-income country with a multiethnic population comprised of three main ethnicities namely Malay (67.4%), Chinese (24.6%) and Indian (7.3%) [14]. Type 2 diabetes is a major health problem in Malaysia and its prevalence continues to rise from 13.4% in 2015 to 18.3% in 2019 [15]. Diabetes is a significant risk factor for cardiovascular disease, which is the main principle cause of death in Malaysia [16]. Based on the Malaysia National Diabetes Registry, 76.2% of patients with type 2 diabetes did not achieved the Malaysian glycaemic treatment target of HbA1c <6.5% and only 21.4 % were on insulin in 2012 [17]. The cost of care of diabetes and its complications was estimated at RM2.04 billion of which almost 70% was borne by the Malaysian government in 2011 [18]. Given the increasing prevalence of type 2 diabetes, more patients would require to consider insulin especially when insulin is now recommended for early treatment [19]. However, the decision to initiate insulin is difficult. Patient faced difficulties in making decision about insulin therapy due to numerous factors such as fear of pain and injection, misconceptions that insulin would caused kidney failure and blindness and the perception that the need for insulin indicates end stage of diabetes [20-23]. Hence, there is a need to support decision making for insulin use to control blood sugar and one way to do this is the use of PDA. PDA enables selection of treatment that is align with patients’ needs and preferences [2] and therefore this may increase their adherence to their chosen treatment and ultimately improve their glycaemic control. Early insulin initiation can prevent early onset of diabetes complications and this in turn may help the Malaysian government to control diabetes care costs.*  *In 2012, a locally developed insulin PDA (https://decisionaid.ohri.ca/AZsumm.php?ID=1558) [8, 26] has been developed to help patients make informed decision on insulin initiation, to promote patient centered care and SDM.* |
| 47. | “…as they were less empowered and rely on others…”: This is an overreach of your results. This was what providers said, not the patients. I think the results don’t do enough to illustrate the patient’s voice in this study. | **Response to reviewer**  We acknowledge that patients’ voice in this study may have been underrepresented. Similar to the justification given above, implementation science generally focuses on understanding and change HCPs’ behaviours as opposed to patient behaviours (Nilsen et al., 2013).  *Nilsen P, Ståhl C, Roback K, et al. Never the twain shall meet? - a comparison of implementation science and policy implementation research. Implementation Science. 2013;8(1):63.  Nevertheless, we recognize the importance of engaging patients in the design and delivery of innovations as their characteristics, beliefs and attitudes can have a significant impact on implementation outcomes. Therefore, in this current, we tried to obtain views on factors influencing PDA implementation by including all relevant stakeholders that include healthcare policy makers, HCPs and patients.  Nonetheless, we have included the lack of patients’ voice in this study as a study limitation.  **Page 30, line 555-557**  **Study limitation**  *Third, the lesser number of patients recruited as compared to HCPs in each clinic might have rendered patients’ voice to be underrepresented.* |
