# Supplementary material for: Factors influencing implementation of an insulin patient decision aid at public health clinics in Malaysia: A qualitative study
Source: PLoS One. 2020 Dec 30;15(12):e0244645. doi: 10.1371/journal.pone.0244645 (PMC7773191; doi:10.1371/journal.pone.0244645)
Supplement: Attachment — Submitted filename: Response to reviewer 2.docx [file pone.0244645.s005.docx]

| **Reviewer** | **Response to reviewer** |
| --- | --- |
| Reviewer #1:  For reasons of clarity I suggest to always talk about perceived factors for implementation, at least in the abstract. | **Response to reviewer**  We have now made changes to some of the texts in the abstract as well as throughout the manuscript to make it clear that this study was on perceived factors for implementation.  Page 2  ***Abstract***  *Background*  *Many patient decision aids (PDAs) are developed in academic settings by academic researchers. Academic settings are different from community clinics where the focus is on clinical work. Thus, research on implementation in community settings will provide insights to effective implementation of PDA in real-world settings. This study explores* ***perceived*** *factors influencing implementation of an insulin patient decision aid (PDA) in five public community health clinics.*  *Methods*  *This study adopted a comparative case study design with a qualitative focus to identify similarities and differences of the potential barriers and facilitators to implementing the insulin PDA across different implementation sites. Focus groups and individual interviews were conducted with 28 healthcare providers and 15 patients from five public community health clinics under the Ministry of Health in Malaysia. The interviews were transcribed verbatim and analysed using the thematic approach.*  *Results*  *Five themes emerged which were time constraints; PDA costs; tailoring PDA use to patient profile; patient decisional role; and leadership and staff motivation. Based on the interviews and drawing on observations and interview reflection notes, time constraints emerged as the common prominent factor that cut across all the clinics, however the themes of tailoring PDA use to patient profile; patient decisional role; leadership and staff motivation contrasted due to the distinct challenges faced by specific clinics. Among clinics from semi-urban areas with more patients from lower education and socio-economic status, patients’ ability to comprehend the insulin PDA and their tendency to rely on their doctors and family to make health decisions were felt could be a prominent barrier to the insulin PDA implementation. Staff motivation appeared to be stronger in most of the clinics where specific time was allocated to diabetes team to attend to diabetes patients and* ***this was felt could be a potential facilitator,*** *however, a lack of leadership might affect the insulin PDA implementation even though a diabetes team is present*  *Conclusions*  *This study found that effective implementation of the insulin PDA across different public health clinics would depend on leadership and staff motivation and, the need to tailor PDA use to patient profile. To ensure successful implementation, implementers should avoid a ‘one size fits all’ approach when implementing health innovations.*  Page 4 line 2  *Therefore, this study aims to identify* ***perceived*** *factors influencing implementation of the insulin PDA in public community clinics in Malaysia*  Page 21, line 755  Discussions  *This study uncovered perceived factors influencing implementation of PDA in public health clinics in Malaysia.* |
|  |  |
| Reviewer #2:  Introduction:  1) Is it possible to shorten the first three paragraphs into one paragraph? It seems we can get to the purpose of your paper more quickly:  PDAs are a means to enhance patient-centered care, for SDM between patient and provider.  Although shown to be effective in improving decision making, implementing PDAs in routine clinical settings can be hampered by xx, xx, and xx. Furthermore, there is limited data on implementing PDAs broadly in Asia. Then you can delete the third paragraph. | **Response to reviewer**  We thank the reviewer for the suggestion. We have now shortened the three paragraphs to one paragraph.  Page 3, line 2-7  Introduction  *Patient decision aids (PDAs) are tools to facilitate shared decision making (SDM) between patient and provider. Although shown to be effective in improving decision making [1], their implementation in routine clinical settings is still lacking [2], hampered by various factors such as time constraints, healthcare professionals’ attitude, patient characteristics, clinic capacity and processes of care and the healthcare environment [2-4]. Furthermore, there is limited data on PDA implementation in Asia.* |
| 2) Thank you for comprehensively answering the questions about diabetes burden in Malaysia and the need for insulin therapy. Some of this may save some space in the introduction if it’s included in the Methods. For example, Lines 25-27 are more about the ‘setting”. I would omit this and consider including it in the “setting” section of the methods. Instead, begin this paragraph with a clear topic sentence: “PDAs may be useful in a place such as Malaysia, particularly for appropriate SDM in diabetic patients.” | **Response to reviewer**  We have made changes to the texts as suggested.  Page 3, line 15-16  Introduction  *PDAs may be useful in a place such as Malaysia, particularly for appropriate SDM in diabetic patients. Type 2 diabetes has risen rapidly in Malaysia*  Page 5, line 11-14  Methods  Settings  *This study was conducted in five public health clinics located in the area of Klang Valley and Selangor, Malaysia; an upper middle-income country with a multiethnic population comprised of three main ethnicities namely Malay (67.4%), Chinese (24.6%) and Indian (7.3%) [23].* |
| 3) Shorten the next three sentences: “Type 2 diabetes has risen rapidly in Malaysia to 18.3% prevalence in 2019, and is a significant factor for cardiovascular disease that is the leading cause of death in Malaysia.” | **Response to reviewer**  We thank the reviewer for the suggestions on the changes in the texts and have revised them as suggested at page xx, line xx.  Page 3, line 16-18  Introduction  *Type 2 diabetes has risen rapidly in Malaysia to 18.3% prevalence in 2019 [8] and is a significant factor for cardiovascular disease that is the leading cause of death in Malaysia [9].* |
| 4) Next: “As nearly three-fourths of Malaysian diabetic patients are unable to achieve glycemic targets (REF), insulin is now recommended for early treatment (REF). However, there are a number of factors and misconceptions that make Malaysian patients reluctant to initiate insulin therapy such as fear of pain and injections, risks for kidney failure, and the perception that insulin therapy indicates end stage diabetes (ref).” | **Response to reviewer**  We thank the reviewer for the suggestions on the changes in the texts and have revised them as suggested.  Page 3, line 18-22  Introduction  *As nearly three-fourths of Malaysian diabetic patients are unable to achieve glycemic targets [10], insulin is now recommended for early treatment [11]. However, there are a number of factors and misconceptions that make Malaysian patients reluctant to initiate insulin therapy such as fear of pain and injections, risks for kidney failure, and the perception that insulin therapy indicates end stage diabetes [12-15].* |
| 5) The next paragraph, some of the information can be cut and added to the Methods. I would focus the introduction to say, “Hence, a PDA for insulin therapy in diabetes has been created in Malaysia (25), available in the local languages to cater to the multiethnic, multilingual Malaysian population.” | **Response to reviewer**  We have revised the texts as suggested.  Page 3 line 22 to page 4, line 2.  Introduction  *Hence, a PDA for insulin therapy in diabetes has been created in Malaysia, available in the local languages (Malay, Chinese, Tamil and English) to cater to the Malaysian population (https://decisionaid.ohri.ca/AZsumm.php?ID=1558) [16]. Although the insulin PDA has been piloted [17], formal implementation and integration for regular use in community clinics in Malaysia have not been conducted.* |
| 6) Line 53-56 “The insulin PDA met…” can be saved for the methods. | **Response to reviewer**  We have removed the sentence, as we do not think it fits with any sections in the Methods.  ~~The insulin PDA met 7/7 for decision aid criteria and 5/8 of reducing risk of bias criteria set by the International Patient Decision Aids Standards Collaboration, which assesses the quality and standards of a PDA [2].~~ |
| 7) Shorten Line 56-61 to: “Although the insulin PDA has been piloted, studies on implementation of the PDA and integration for regular use in community clinics in Malaysia have not been conducted.” | **Response to reviewer**  We have changed the texts as suggested.  Page 3 line 25 to page 4, line 2.  Introduction  *Although the insulin PDA has been piloted [17], formal implementation and integration for regular use in community clinics in Malaysia have not been conducted.* |
| 8) Then I would jump directly to the final paragraph: “Therefore, this study aims to identify factors influencing…” | **Response to reviewer**  We have changed the texts as suggested  Page 4, line 2-6  Introduction  *Therefore, this study aims to identify perceived factors influencing implementation of the insulin PDA in public community clinics in Malaysia. The findings of this study hope to inform future development of common and contextual implementation strategies to incorporate PDA use into standard clinical practice in community clinics under the same organization.* |
| 9) I am a little confused with the mention of “prospectively” in line 73; from the study it seems like the review was done retrospectively after the PDA had been introduced in clinics? I note the comment to the other reviewer to clarify this, but to me, it seems a prospective study of this type would be a planned evaluation AFTER the implementation of a PDA, but the study design does not seem that it is so structured. | **Response to reviewer**  We have now removed the word “prospectively” and replaced it with the word “perceived” for better clarity.  Page 4, line 2  *Therefore, this study aims to identify* ***perceived*** *factors influencing implementation of the insulin PDA in public community clinics in Malaysia.* |
| Methods:  10) Lines 81-86, the first three sentences can be shortened to one. | **Response to reviewer**  We have removed some of the sentenced and revised the texts to the following:  Page 4, line 10-13  *This study adopted a comparative case study design with a qualitative focus [18, 19]. This study design was chosen because it allowed identification of similarities and differences of potential barriers and facilitators to implementing the insulin PDA across different as well as within implementation sites.* |
| 11) Lines 88-93 are quite repetitive; can you shorten to maybe 1-2 sentences? | **Response to reviewer**  We have removed some of the sentenced and revised the texts to the following:  Page 4, line 10-13  *Study design and participants*  *This study adopted a comparative case study design with a qualitative focus [18, 19]. This study design was chosen because it allowed identification of similarities and differences of potential barriers and facilitators to implementing the insulin PDA across different as well as within implementation sites.* |
| 12) Thank you for clarifying in-depth interviews, but for the wider audience, “individual” is not needed, I know this was included to help me; it is now clear because I know who will be interviewed (clinic managers). But here is some suggestion to state the most important information succinctly: “In-depth interviews (IDI) with clinic managers and focus group discussions (FGD) with HCPs and patients were conducted. All components were conducted to ensure quality data was collected; clinic managers were not included in FGD with HCPs for fear that this would change power dynamics in the group. FGD with HCPs were separate from patients, so that FGDs would include participants that are peers to enable richer and more robust findings.” | **Response to reviewer**  We have now removed the word “individual” and revised the texts based on the suggestion  Page 4, line 15-21  Study design and participants  *In this study, in-depth ~~individual~~ interviews (IDI) with clinic managers and focus group discussions (FGD) with HCPs and patients were conducted. All components were conducted to ensure quality data was collected; clinic managers were not included in FGD with HCPs for fear that this would change power dynamics in the group. FGD with HCPs were separate from patients, so that FGDs would include participants that are peers to enable richer and more robust findings.* |
| 13) Please note, sometimes plural forms of the acronyms are used, e.g., “IDIs”, other times not; just a note to make sure the authors are consistent. | **Response to reviewer**  We thank the reviewer for pointing this. We have now made sure the ‘IDIs’ and ‘FGDs’ are consistent throughout the manuscript. |
| 14) I would try to include lines 62-71 in the “Settings” section of the Methods instead of the introduction. I would take this paragraph and include it as a second paragraph in your “settings” section: “These five public health clinics fall under the Malaysia Ministry of Health. Diabetes patients in Malaysia are largely seen…work culture.” | **Response to reviewer**  We have revised the texts as suggested by the reviewer.  Page 5, line 11-21  Method  *This study was conducted in five public health clinics located in the area of Klang Valley and Selangor, Malaysia; an upper middle-income country with a multiethnic population comprised of three main ethnicities namely Malay (67.4%), Chinese (24.6%) and Indian (7.3%) [3]. These five clinics fall under the Malaysia Ministry of Health [4]. Diabetes patients in Malaysia are largely seen at these clinics where insulin initiation is mainly conducted due to the availability of the resources and the fact that insulin treatment are subsidized. In the public community clinics, implementation of innovations are often conducted using the top-down approach without taking into consideration the needs of the frontline staff who puts the intervention into practice. It should be noted that while the community clinics are under the same organization, the clinics vary in terms of locality, leadership, patient population and work culture.* |
| 15) I actually would take what has been written under “study participants” and add it to the end of the “Study design” section, and retitle the study design section, “Study design and participants.” Then it becomes much clearer who is included in the IDI vs FGD. Also, be careful to say that they are policy maker OR clinic managers, but using too many categories may be confusing for the reader. In the results you use mainly the word “health policymaker.” | **Response to reviewer**  We have now place “Study participants” under “Study design” and retitled the section to “Study design and participants” as suggested.  We have also change the term ‘policy maker’ to ‘clinic manager’ throughout the manuscript.  Page 4 line 10 to page 5 line 8  **Study design and participants**  *This study adopted a comparative case study design with a qualitative focus [18, 19]. This study design was chosen because it allowed identification of similarities and differences of potential barriers and facilitators to implementing the insulin PDA across different as well as within implementation sites.*  *In this study, in-depth interviews (IDIs) with clinic managers and focus group discussions (FGDs) with HCPs and patients were conducted. All components were conducted to ensure quality data was collected; clinic managers were not included in FGDs with HCPs for fear that this would change power dynamics in the group. FGDs with HCPs were separate from patients, so that FGDs would include participants that are peers to enable richer and more robust findings. However, for participants who were unable to participate in FGDs, IDIs were conducted to accommodate their schedules.*  *The participants of this study were purposively sampled and consisted of 1) clinic managers who have the authority to decide which health intervention should be implemented in the clinic, 2) HCPs (family medicine specialist, medical officer, diabetes educator, staff nurse, pharmacist) who are involved in advising patients about starting insulin and 3) patients with type 2 diabetes who have been seeking treatment in the clinics and advised to, or, are currently using insulin. In literature surrounding SDM and PDA implementation, patient's perspectives on implementation processes are often not reported even though they are the end-users of the innovation. Patient can contribute such as by informing from who and how they want the innovation to be delivered to them [20-22]. Patients are included in this study to obtain a more comprehensive finding on potential factors that could influence the insulin PDA implementation is obtained.* |
| 16) Table 2, and the Results: the themes can be shortened. Can there be one or two words for “Time, patient load, lack of manpower?” “Cost to print PDA” can be just “PDA costs”? The other themes are appropriate I feel. | **Response to reviewer**  We have now change the texts “Time, patient load, lack of manpower?” to “Time constraints” and “Cost to print PDA” to “PDA costs” throughout the manuscript.  Page 15, line 244  Theme 1: Time constraints  Page 15, line 238  Data analysis uncovered five themes related to perceived factors that could influence the implementation of the insulin PDA in the five clinics and they were: **1) time constraints, 2) PDA costs,** 3) tailoring PDA use to patient profile, 4) patient decisional role, and 5) leadership and staff motivation.  Page 16, line 293  Theme 2: PDA costs |
| Results:  18) Table 3 is tricky. I find tables in qualitative data are very tricky. Theoretically, the case can be argued that static indicators, such as age, HCP duration, patient durations, are not needed to be so strictly summarized, as these are not going to be “representative” in a quantitative sense.  As a reviewer, I feel IDI and FGD at the different clinic is helpful, but I would also like to see the breakdown of FGD for HCPs vs patients. The way the columns and rows are set up, the ethnicity, education level or exactly what participant is unclear.  Again, as a reviewer, it would be nice to see the ethnicity and education level for FGDs, HCPs and patients separately. In the end, this may be easier to present as text instead of a table. | **Response to reviewer**  We have now revised the table to make the breakdown of ethnicity and education level for HCPs and patients in the FGDs clearer.  Page 13  Table 3: Participants’ socio-demographic information by clinic |
| 19) Lines 241-242 now a bit unclear because I originally requested to take away the numbers. Sorry! | **Response to reviewer**  We have added the numbers to make the themes appear clearer.  Page 15, line 237-238  Results  Data analysis uncovered five themes that were perceived as potential factors that could influence the implementation of the insulin PDA in the five clinics and they were: 1) time constraints, 2) PDA costs, 3) tailoring PDA use to patient profile, 4) patient decisional role, and 5) leadership and staff motivation. |
| Discussion  21) This is the section that may need some significant revisions. The length of this section actually suggests to me that although the Results are stronger, the Discussion includes information that likely should be in the Results.  22) The first paragraph, from lines 399 until 409, is very strong. After this point the discussion gets very wordy and confused. Aim to have a 5-paragraph discussion. Outline the main points to convey to the reader. Be very strict on each paragraph.  Make sure that one of these paragraphs is the limitations. Anything that is special and important in the discussion that does not relate to implication or how this study fits with other literature, should be placed back in the results. Avoid restating the results in the Discussion.  23) The paragraphs currently go back and forth between implications of this study for PDAs in general, and implications from this study about PDAs for insulin implemented in Malaysia. Then there is discussion about what to do for enhancing SDM, as well. This makes for a discussion that is quite hard to follow. | **Response to reviewer**  We have now revised the Discussion section and reduced the length of the Discussions to a 5-paragraph discussion including limitations. We first provide a general overview of the findings in terms of the micro (patient population, leadership and staff motivation) and macro (provision of resources) contextual factors that can affect the insulin PDA implementation. In the subsequent paragraphs, each of the factors starting from micro to macro factors was discussed. We have removed some of the results in this section. We have also revised the texts in the way that the findings of this study can inform implementation of PDAs in general.  *Page 21 line 755 to page 25 line 751*  *Discussion*  *This study uncovered perceived factors influencing implementation of PDA in public health clinics in Malaysia. The findings highlighted that besides looking into macro level of implementation such as cost and provision of resources, healthcare authorities should take consideration of the micro contextual factors that affect implementation such as a clinic’s patient population, leadership and staff motivation at specific clinic settings. There is a need for implementation strategies to be tailored to address the contextual needs at each implementation site as a one-size-fits-all approach may not be effective for implementation [28].*  *The findings of this study pointed to the unique implementation challenges that were prominent to some clinics in terms of their patient population such as patients’ ability to comprehend the information in the PDA due to low literacy, language barrier and those who tend rely on others to make health decisions for them. When implementing PDAs, healthcare authorities need to be aware of the barriers faced by individual clinics in relation to their context and specific strategies have to be employed. The use of video images in PDAs, having HCPs to deliver PDAs to patients in a straightforward, plain language, tailoring the level of information to individual patients’ needs and in the context of clinical care may help to facilitate patients’ understanding of the information in the PDAs among those with low literacy [21, 29-31]. To address communication language barrier between HCP and patient during PDA discussions, presence of multi-ethnic staff in a clinic was noted to be solution in this study and healthcare authority can consider providing staff of different ethnicities to clinics, which faced the same barrier. To address the barrier of patients relying on HCPs to make health decisions, efforts to increase patients’ awareness on being more involved in their health care and self-efficacy in making health decision, as well as participation in SDM are warranted. One study have shown that an intervention to reduce patients’ unvoiced needs by getting patients to note down issues to be discussed with doctors prior to their clinical encounter to be effective [32] and may be adopted to faciliate PDA implementation.*  *This study also found that there is a variation in terms of leadership and staff motivation for diabetes promotion among the clinics. Getting support from clinic leadership and clinical champion is one of the key facilitators for effective PDA and SDM implementation [2, 33, 34]. To increase staff motivation to use the PDA, there is a need to make HCPs to recognize and believe the benefits of practicing SDM and use of PDAs. At the healthcare system level, incentivising the use of PDA may be the key to motivating HCPs to be involved in the implementation. One example is such as making PDA use as an alternative means of informed consent to protect HCPs from ‘failure to inform’ lawsuit [35]. At the broader level of PDA implementation is the need to inculcate SDM culture and practice and HCPs need to be taught on the concept of SDM and PDA. HCPs need to understand their role in SDM in terms of providing quality information to patients and supporting patients in their deliberation of the treatment option [36]. SDM is generally a foreign concept in Malaysia in that SDM activities only began in 2010 [37]. While the Ministry of Health Malaysia and the Malaysian Medical Council has encourage doctors to perform SDM-related activities such as providing patients with accurate and timely information, discuss treatment limitation and risks, and collaborate with patient in selection of treatment since 1990s [37-39], patient involvement in healthcare decision making in Malaysia was still found to be lacking as there is no formal training and education, laws, regulations and health policies that supports SDM [40]. SDM is not taught explicitly in the medical undergraduate and postgraduate curriculum [37]. To implement SDM and PDA effectively, HCP training is warranted. Studies have reported the lack of training to be contributing factors to lack of engagement of patients in SDM and proper use of PDAs [41, 42]. The benefits of training are well evidenced [43, 44] and it should be a pre-requisite for SDM and PDA implementation [45].*  *This study found that provision of resources such as manpower and funding by the Ministry of Health is not adequate for the clinics’ current operation. This can have significant impact on PDA implementation or even implementation of other health innovations in general. It was found that the diabetes educators provided in the clinics had to perform other duties, which were not relevant to their job scope. Such would be a waste and inefficient use of the diabetes educator’s expertise to help in the delivery of the PDA to patients. Involving diabetes educator in the implementation of PDA can help to avoid dependence on doctors in using the PDA with patients during consultation. Given that manpower resources provided by the Ministry of Health is already stretched, increasing manpower is not likely to be possible. Strategies similar to increasing existing staff motivation to be involved in the PDA implementation as described above can be adopted. In terms of the issue of funding, efforts are needed to obtain buy-in from the Ministry of Health to invest in PDA implementation so that specific allocation of the national healthcare funding can be provided. There is a need to show healthcare authority on the advantages of PDA in terms of improved patient care [1] as well reduced healthcare costs [46]. More local evidences are needed on the positive impact of the insulin PDA implementation to clinic services as well as patient health outcomes to obtain the clinics’ higher authorities buy-in to prioritise the insulin PDA implementation. An alternative to address the cost of PDA implementation is the use of web-based PDAs, which preclude the need for printing costs and can be access freely by patients.* |
