# Supplementary material for: Factors influencing implementation of an insulin patient decision aid at public health clinics in Malaysia: A qualitative study
Source: PLoS One. 2020 Dec 30;15(12):e0244645. doi: 10.1371/journal.pone.0244645 (PMC7773191; doi:10.1371/journal.pone.0244645)
Supplement: Attachment — Submitted filename: Response to reviewer - Revision 3.docx [file pone.0244645.s006.docx]

| **Reviewers’ comments** | **Response to reviewer** |
| --- | --- |
| Abstract:  1. Line 8: PDA previously defined, so does not need to spell it out here. | **Response to reviewer**  We thank the reviewer for pointing this out. We have revised the texts.  Page 2, line 8  *…factors influencing implementation of an insulin* ***PDA*** *in five public health…* |
| 2. Line 14: omit “implementation” | **Response to reviewer**  We have now revised the sentence as suggested by the reviewer.  Page 2, line 13  *…insulin PDA across different sites.* |
| 3. Line 15: “public community health clinics” but I prefer what is said in the methods, “public health clinics”, which would be consistent with the methods, too. Also need to make sure “public health clinics” is used consistently throughout the paper. | **Response to reviewer**  We now use ‘public health clinics’ and have made sure this is consistent throughout the manuscript. |
| 4. Line 20: can number these themes again as they are in the results. | **Response to reviewer**  We have numbered the themes as suggested.  Page 2, line 19-20  *Five themes emerged which were: 1) time constraints; 2) PDA costs; 3) tailoring PDA use to patient profile; 4) patient decisional role; and 5) leadership and staff motivation.* |
| 5. Line 23: omit “the themes of” | **Response to reviewer**  We have removed the texts as suggested.  Page xx, line xx  *Based on the interviews and drawing on observations and interview reflection notes, time constraints emerged as the common prominent factor that cut across all the clinics, however, tailoring PDA use to patient profile; patient decisional role; leadership and staff motivation varied due to the distinct challenges faced by specific clinics.* |
| 6. Line 24: change “contrasted” to “varied” | **Response to reviewer**  We have changed the word ‘contrasted’ to ‘varied’  Page 2, line 20-24  *Based on the interviews and drawing on observations and interview reflection notes, time constraints emerged as the common prominent factor that cut across all the clinics, however, tailoring PDA use to patient profile; patient decisional role; leadership and staff motivation* ***varied*** *due to the distinct challenges faced by specific clinics.* |
| 7. Line 26: should read “limited education and lower socio-economic status”—I recommend using this phrase in the other sections as well. | **Response to reviewer**  We have changed the text as suggested throughout the manuscript  Page 2, line 25  *Among clinics from semi-urban areas with more* ***patients from limited education and lower socio-economic status****, patients’ ability to comprehend the insulin PDA and their tendency to rely on their doctors and family to make health decisions were felt could be a prominent barrier to the insulin PDA implementation.*  Page 17, line 287-288  *Among the clinics from semi-urban areas where many* ***patients were reported to have limited education and lower socio-economic status*** *(Clinic D and E),…*  Page 18, line 326  *compared to clinics where many patients had* ***limited education*** *(Clinic D and E).* |
| 8. Change “could” to “to” in lines 28 and 30. | **Response to reviewer**  We have now changed the word ‘could’ to ‘to’  Page 2, line 27  *health decisions were felt* ***to*** *be a prominent barrier to the insulin PDA implementation.* |
| 9. Conclusions are a bit odd; in the results the authors state that the most important one is time constraints but this isn’t reflected in the conclusions? As mentioned below, need to make sure Conclusions in the Discussion match the Introduction. | **Response to reviewer**  We have revised the study conclusion and have included ‘time constraint’ in the conclusion. We have also made sure the conclusion in the Discussion is similar to the conclusion in the Abstract.  Page 2 and page 25  *Conclusions*  *This study found* ***time constraint as a major potential barrier for PDA implementation*** *and effective implementation of the insulin PDA across different public health clinics would depend on leadership and staff motivation and, the need to tailor PDA use to patient profile. To ensure successful implementation, implementers should avoid a ‘one size fits all’ approach when implementing health innovations.* |
| Introduction:  10. Line 25 pg 3, “Although the insulin PDA” can be a new paragraph. | **Response to reviewer**  We have moved the sentence to a new paragraph at page 4, line 4. |
| Methods:  12. HCPs is defined in line 25 but can be defined when it is first mentioned earlier? | **Response to reviewer**  We have defined HCP when it is first mentioned at page 4 line 19 and used the acronym at subsequent pages throughout the manuscript. |
| 13. Table 1: I don’t think the columns need to be divided into multiple rows for each subheading. E.g., I think it’s find to have “Patient profile” where for clinic A there is no border between predominantly Chinese and Indian, middle to high income group, high education level. Same for the following subheadings and across the columns. | **Response to reviewer**  We have now revised Table 1 based on the suggestions given at page 7. |
| 14. Lines 230-231: not needed to include the degrees, but the second sentence in line 231 is sufficient. | **Response to reviewer**  We have now removed the degrees.  Page 8, line 229-230  *The researchers of this study are WTT, YKL, NCJ and PYL. WTT is a PhD student while YKL, CJN and PYL are lecturers.* |
| Discussion  15. This can be shortened. The main bias is “social desirability” reflected by the manager interviewed as well as the mangers’ selection of HCPs to participate. But I don’t think this level of detail is necessary.  “Social desirability bias may have been present with participants who were favorable to either the use of the PDAs or those that wanted to provide a favorable view of their clinic.” This and the fifth limitation can be kept and grouped together as they are both answered by the last sentence. The fourth limitation is a good one, but add that you included patients where most of the other literature doesn’t look at this important participant group. I would omit limitations two and three. | **Response to reviewer**  We thank the reviewer for the suggestions on how to improve the reporting of the study limitations. We have now revised the study limitations as suggested.  Page 24, line 753-737  **Study limitations**  *This study has a few limitations. Social desirability bias may have been present with participants who were favorable to either the use of the PDAs or those that wanted to provide a favorable view of their clinic. In addition, findings from the data analysis were not provided to the participants for feedback. However, the researcher ensured that the data were accurately interpreted by crosschecking the analysis of the findings to interview reflections and memos that were jotted down at the end of each interview. Lastly, the few patients recruited as compared to HCPs in each clinic might have rendered patients’ voice to be underrepresented. Nevertheless, this study provided insights into patients’ perspectives that were scarcely reported in literature surrounding PDA implementation.* |
| 16. Make sure the conclusions in the Discussion are the same as those in the abstract. | **Response to reviewer**  We have now made sure the conclusion in the Discussion is same with the conclusion in the Abstract. |
